# Care for adults with COVID‐19: living guidelines from the National COVID‐19 Clinical Evidence Taskforce

**DOI:** 10.5694/mja2.51718

**Published:** 2022-09-23

**Authors:** Heath White, Steve J McDonald, Bridget Barber, Joshua Davis, Lucy Burr, Priya Nair, Sutapa Mukherjee, Britta Tendal, Julian Elliott, Steven McGloughlin, Tari Turner

**Affiliations:** ^1^ Cochrane Australia Monash University Melbourne VIC; ^2^ QIMR Berghofer Medical Research Institute Brisbane QLD; ^3^ John Hunter Hospital Newcastle NSW; ^4^ The University of Newcastle Newcastle NSW; ^5^ Mater Hospital Brisbane Brisbane QLD; ^6^ Mater Research Institute University of Queensland Brisbane QLD; ^7^ St Vincent's Hospital Sydney Sydney NSW; ^8^ Adelaide Institute for Sleep Health Adelaide SA; ^9^ The Alfred Hospital Melbourne VIC; ^10^ Monash University Melbourne VIC

**Keywords:** Public health, Primary health care, COVID‐19, Antiviral agents

## Abstract

**Introduction:**

The Australian National COVID‐19 Clinical Evidence Taskforce was established in March 2020 to maintain up‐to‐date recommendations for the treatment of people with coronavirus disease 2019 (COVID‐19). The original guideline (April 2020) has been continuously updated and expanded from nine to 176 recommendations, facilitated by the rapid identification, appraisal, and analysis of clinical trial findings and subsequent review by expert panels.

**Main recommendations:**

In this article, we describe the recommendations for treating non‐pregnant adults with COVID‐19, as current on 1 August 2022 (version 61.0). The Taskforce has made specific recommendations for adults with severe/critical or mild disease, including definitions of disease severity, recommendations for therapy, COVID‐19 prophylaxis, respiratory support, and supportive care.

**Changes in management as a result of the guideline:**

The Taskforce currently recommends eight drug treatments for people with COVID‐19 who do not require supplemental oxygen (inhaled corticosteroids, casirivimab/imdevimab, molnupiravir, nirmatrelvir/ritonavir, regdanvimab, remdesivir, sotrovimab, tixagevimab/cilgavimab) and six for those who require supplemental oxygen (systemic corticosteroids, remdesivir, tocilizumab, sarilumab, baricitinib, casirivimab/imdevimab). Based on evidence of their achieving no or only limited benefit, ten drug treatments or treatment combinations are not recommended; an additional 42 drug treatments should only be used in the context of randomised trials. Additional recommendations include support for the use of continuous positive airway pressure, prone positioning, and endotracheal intubation in patients whose condition is deteriorating, and prophylactic anticoagulation for preventing venous thromboembolism. The latest updates and full recommendations are available at www.covid19evidence.net.au.

The severe acute respiratory syndrome coronavirus 2 (SARS‐CoV‐2) quickly drew the attention of virologists, epidemiologists, and infectious disease experts after its identification in Wuhan (China) in December 2019. The speed with which the virus was spreading and the imminent challenge to global health as the pathogen of coronavirus disease 2019 (COVID‐19) were evident even before the World Health Organization declared the outbreak a pandemic in March 2020.^1^

The novelty of both the virus and the disease meant that very little information was initially available to guide decisions about treating and preventing illness. As the pandemic spread, there was an unprecedented push by investigators around the world to rapidly plan, conduct, and publish the results of clinical studies to provide such information. Effectively managing the subsequent explosion in evidence required consideration of two factors. Firstly, a process was needed to rapidly incorporate evidence into guidelines to ensure that recommendations remained up to date. Secondly, a single voice providing consistent advice to clinicians, rather than potentially conflicting advice from multiple bodies, was needed. The Australian National COVID‐19 Clinical Evidence Taskforce (https://covid19evidence.net.au), originally comprising 32 organisations representing health care workers caring for people with COVID‐19, was established to achieve these goals in Australia.

In this article, we describe the recommendations for treating non‐pregnant adults with COVID‐19 in Australia, as current on 1 August 2022 (version 61.0). Previous Taskforce publications have included recommendations specific for children and adolescents,^2^ for women who are pregnant or have recently given birth,^3^ and for older people and people requiring palliative care.^4^

## Methods

In view of the unprecedented global research volume and the rapidly evolving evidence base, the Taskforce adopted a living approach to guideline development. Living guidelines enable rapid identification and translation of research findings into recommendations, ensuring that advice reflects current knowledge.^5^, ^6^ Prior to the COVID‐19 pandemic, Australian Living Evidence Consortium members had developed and continuously improved the methods of evidence‐based living guideline production,^7^, ^8^ and the ability to use the knowledge and systems developed during this process has been crucial to the success of the Taskforce.

The Taskforce uses a two‐stage, high throughput process to ensure rapid updating of recommendations as new evidence becomes available, while maintaining scientific rigour and transparency. We use efficient methods for identifying, analysing, and reviewing evidence, then apply a streamlined process for developing, approving, and publishing new and updated recommendations. As a result, a guideline can be updated and published within days of the publication of the findings of a major study, considerably more rapid than for a traditional guideline.^9^ We have previously published details of the methods used by the Taskforce.^10^

### Strength of recommendations

The Taskforce uses the Grading of Recommendations Assessment, Development and Evaluation (GRADE) framework^11^ to determine the strength and direction of its recommendations. We primarily base recommendations regarding medications on the findings of randomised controlled trials, but data from observational studies are occasionally considered. Following analysis of the evidence, the relevant Taskforce expert panel develops a strong or conditional recommendation for or against an intervention, based on the balance of benefits and harms, certainty of evidence, and other considerations, including resource use, equity, and acceptability. The importance of each outcome is ranked and the threshold required to reach clinical significance defined. Certainty of evidence is determined for each outcome as high, moderate, low, or very low by applying a set of established criteria, including risk of bias, inconsistency, indirectness, imprecision, and publication bias. If sufficient high quality evidence is not available, the panel may develop a consensus recommendation based on expert opinion. Alternatively, insufficient evidence for determining the benefits and harms of a treatment may lead to an “only in research” recommendation; that is, the treatment should not be used outside randomised trials with appropriate ethics approval.

### Definition of disease severity

Definitions of disease severity in people with COVID‐19 have not always been consistent across trials. Further, trial publications frequently do not use the terms “mild”, “moderate”, “severe”, and “critical” to define the included patient group, instead providing clinical data such as respiratory rate, blood oxygen saturation, and presence of lung infiltrates. An important task for the Taskforce was to develop a consensus recommendation for defining levels of disease severity for adults (Supporting Information, table 1).

## Recommendations

### Disease‐modifying treatments

The paucity of direct evidence initially available to the Taskforce is exemplified by the single consensus recommendation regarding therapeutic agents for COVID‐19 in version 1.0 of the guideline (3 April 2020): “For patients with COVID‐19 illness, only administer antiviral medications or other disease‐modifying treatments in the context of clinical trials with appropriate ethical approval”.

We have now expanded this consensus recommendation to 77 adult‐specific recommendations based on findings from more than 170 primary studies reported to 1 August 2022. Most treatments have been assigned “only in research” recommendations (because of insufficient evidence) or “do not use” recommendations (sufficient evidence that treatment is of limited or no benefit).

### Adults who require supplemental oxygen

The Taskforce supports the use of six therapeutic agents for treating people with severe or critical COVID‐19 (ie, those who require supplemental oxygen; Box 1).

Box 1 Drug treatments recommended for use in people with severe or critical coronavirus disease 2019 (COVID‐19) (ie, who require supplemental oxygen)^*^

**Table:** 

Drug treatment	Category	Recommendation
Corticosteroids (systemic)	Recommended	Use intravenous or oral dexamethasone for up to ten days (or another acceptable regimen) in adults who require supplemental oxygen (including mechanically ventilated patients).
	Conditional recommendation against	Do not routinely use dexamethasone (or other systemic corticosteroid) to treat COVID‐19 in adults who do not require supplemental oxygen.
Remdesivir	Conditional recommendation	Consider using remdesivir in adults who require supplemental oxygen but not non‐invasive or invasive ventilation.
	Not recommended	Do not use remdesivir in adults hospitalised with COVID‐19 who require non‐invasive or invasive ventilation.
Tocilizumab	Conditional recommendation	Consider using tocilizumab in adults who require supplemental oxygen, particularly when there is evidence of systemic inflammation.
Sarilumab	Conditional recommendation	Consider using sarilumab in adults who require high‐flow oxygen, non‐invasive ventilation, or invasive mechanical ventilation.
Baricitinib	Conditional recommendation	Consider using baricitinib in adults hospitalised with COVID‐19 who require supplemental oxygen.
Casirivimab/imdevimab	Conditional recommendation	Consider using casirivimab/imdevimab in SARS‐CoV‐2 antibody‐seronegative adults hospitalised with moderate to critical COVID‐19.
	Not recommended	Do not use casirivimab/imdevimab in SARS‐CoV‐2 antibody‐seropositive adults hospitalised with moderate to critical COVID‐19.

SARS‐CoV‐2 = severe acute respiratory syndrome coronavirus 2.

* Status: 1 August 2022. For full recommendations, see Supporting Information, table 2.

Systemic corticosteroid treatment is the only treatment strongly (rather than conditionally) recommended, initially based on the findings of a World Health Organization meta‐analysis of randomised controlled trials (7184 participants in nine studies).^12^ The Taskforce recommends systemic corticosteroids for adults who require supplemental oxygen, and conditionally recommends against using them in adults who do not require supplemental oxygen.

Remdesivir was previously conditionally recommended for adults with moderate to critical COVID‐19. Following the publication of results by the WHO Solidarity Trial Consortium,^13^ the Taskforce applied the Instrument for assessing the Credibility of Effect Modification Analyses (ICEMAN)^14^ and determined that it was appropriate to stratify trial results by disease severity. As a result, the conditional recommendation for the use of remdesivir was retained for patients who are hospitalised and require supplemental oxygen but not ventilation (six studies, 6904 participants^15^, ^16^, ^17^, ^18^, ^19^, ^20^), but the Taskforce recommends against its use in patients who are hospitalised and require ventilation (four studies, 1332 participants^16^, ^17^, ^19^, ^20^).

Subsequently, three immunomodulators have been reported to reduce mortality risk in patients who require supplemental oxygen, and the Taskforce conditionally recommends their use in these patients. Tocilizumab (eleven studies, 7221 participants^21^, ^22^, ^23^, ^24^, ^25^, ^26^, ^27^, ^28^, ^29^, ^30^, ^31^) and sarilumab (seven studies, 3668 participants^32^, ^33^, ^34^, ^35^, ^36^, ^37^, ^38^), are monoclonal antibodies against the interleukin‐6 receptor; baricitinib (four studies, 10 815 participants^39^, ^40^, ^41^, ^42^) is a Janus kinase (JAK) inhibitor. In addition, two studies^43^, ^44^ reported a benefit from casirivimab/imdevimab (Ronapreve; monoclonal antibodies against the SARS‐CoV‐2 spike protein) in adult inpatients seronegative for SARS‐CoV‐2 antibodies (3673 patients), but not in patients seropositive at baseline (7202 patients).

### Adults who do not require supplemental oxygen

Prior to 26 August 2021, there were no treatment options for preventing disease progression in people with mild COVID‐19 (ie, those who do not require supplemental oxygen). Almost eighteen months after the inception of the Taskforce and 64 recommendations regarding 57 different treatments, sotrovimab (Xevudy) received the first conditional recommendation, for use in people with mild COVID‐19 at high risk of disease progression (one study, 1057 participants^45^). The following month, we published a conditional recommendation for the use of inhaled budesonide in people with mild COVID‐19 and one or more risk factors for disease progression. The Taskforce subsequently considered budesonide and ciclesonide sufficiently similar to justify pooling trial data, resulting in a single consensus recommendation for both inhaled corticosteroids (five studies, 2668 participants^46^, ^47^, ^48^, ^49^, ^50^).

At the beginning of 2022, the Taskforce published several additional recommendations regarding treatments for people with mild COVID‐19 and one or more risk factors for disease progression. Facilitated by early access to confidential Therapeutic Goods Administration clinical study reports, the Taskforce conditionally recommended casirivimab/imdevimab (Ronapreve; three studies, 5063 participants^51^, ^52^), the antiviral agents molnupiravir (Lagevrio; one study, 1433 participants^53^) and nirmatrelvir/ritonavir (Paxlovid; one study, 2246 participants^54^), and the anti‐spike monoclonal antibodies regdanvimab (Regkirona; two studies, 1629 participants^55^, ^56^) and tixagevimab/cilgavimab (Evusheld; one study, 903 participants^57^). More recently, findings of a clinically significant reduction in the hospitalisation of people with mild illness treated with the antiviral agent remdesivir (Veklury) have been published (one study, 562 participants^58^).

The inclusion and exclusion criteria of the cited studies were similar. With few exceptions, each included adult outpatients who were not vaccinated against SARS‐CoV‐2 and had one or more risk factors for disease progression. In addition, most studies included only small numbers of immunosuppressed patients. Most of our conditional recommendations are therefore accompanied by a consensus recommendation specific to people considered most likely to benefit from treatment but for whom there is little or no direct evidence available, such as immunocompromised people and people partially vaccinated against SARS‐CoV‐2 and considered to be at high risk of progression because of their age and risk factors (Box 2).

Box 2 Drug treatments recommended for use in people with mild coronavirus disease 2019 (COVID‐19) (ie, who do not require supplemental oxygen)*

**Table:** 

Treatment	Category	Recommendation
Casirivimab/imdevimab	Conditional recommendation	• Consider casirivimab/imdevimab within seven days of symptom onset for adults who do not require supplemental oxygen and have one or more risk factors for disease progression. • When infection with Omicron BA.1, BA.2, BA.4 or BA.5 is confirmed or likely, use of casirivimab/imdevimab should only be considered if other treatments are not suitable or available.
Corticosteroids (inhaled)	Conditional recommendation	• Consider inhaled corticosteroids (budesonide or ciclesonide) within 14 days of symptom onset for adults who do not require supplemental oxygen and have one or more risk factors for disease progression.
Molnupiravir (Lagevrio)	Consensus recommendation	• Consider molnupiravir within five days of symptom onset for unvaccinated adults who do not require supplemental oxygen and have one or more risk factors for disease progression if other treatments (such as remdesivir or nirmatrelvir/ritonavir) are not suitable or available. • Within this group, decisions about the appropriateness of molnupiravir treatment should be based on the individual’s risk of severe disease, including their age, presence of multiple risk factors, and vaccination status (including number of doses and time since last dose or most recent SARS‐CoV‐2 infection).
	Consensus recommendation	• In addition to unvaccinated adults at risk of progression, also consider molnupiravir within five days of symptom onset for adults who do not require supplemental oxygen and are immunocompromised, or who are at particularly high risk of severe disease because of advanced age and multiple risk factors, AND other treatments (such as remdesivir or nirmatrelvir/ritonavir) are not suitable or available.
Nirmatrelvir/ritonavir (Paxlovid)	Conditional recommendation	• Consider nirmatrelvir/ritonavir within five days of symptom onset in unvaccinated adults^†^ who do not require supplemental oxygen and have one or more risk factors for disease progression. • Within this group, decisions about the appropriateness of nirmatrelvir/ritonavir treatment should be based on the individual’s risk of severe disease, including their age, presence of multiple risk factors, and vaccination status (including number of doses and time since last dose or most recent SARS‐CoV‐2 infection).
	Consensus recommendation	• In addition to unvaccinated adults at risk of progression, also consider nirmatrelvir/ritonavir within five days of symptom onset for adults who do not require supplemental oxygen and are immunocompromised, or are at particularly high risk of severe disease because of advanced age and multiple risk factors.
Regdanvimab (Regkirona)	Conditional recommendation	• Consider regdanvimab within seven days of symptom onset for unvaccinated adults^‡^ who do not require supplemental oxygen and have one or more risk factors for disease progression. • Within this group, decisions about the appropriateness of regdanvimab treatment should be based on the individual’s risk of severe disease, including their age, multiple risk factors, SARS‐CoV‐2 vaccination status, and time since vaccination. • When infection with Omicron BA.1, BA.2, BA.4 or BA.5 is confirmed or likely, regdanvimab should only be considered if other treatments are not suitable or available.
	Consensus recommendation	• In addition to unvaccinated adults at risk of progression, also consider regdanvimab within seven days of symptom onset for adults who do not require supplemental oxygen and are immunocompromised, or are at particularly high risk of severe disease because of advanced age and multiple risk factors. • When infection with Omicron BA.1, BA.2, BA.4 or BA.5 is confirmed or likely, use of regdanvimab should only be considered if other treatments are not suitable or available.
Remdesivir (Veklury)	Conditional recommendation	• Consider remdesivir within seven days of symptom onset in unvaccinated adults^†^ who do not require supplemental oxygen and have one or more risk factors for disease progression. • Within this group, decisions about the appropriateness of remdesivir treatment should be based on the individual’s risk of severe disease, including their age, presence of multiple risk factors, and vaccination status (including number of doses and time since last dose or most recent SARS‐CoV‐2 infection).
	Consensus recommendation	• In addition to unvaccinated adults at risk of progression, also consider remdesivir within seven days of symptom onset for adults who do not require supplemental oxygen and are immunocompromised, or are at particularly high risk of severe disease because of advanced age and multiple risk factors.
Sotrovimab (Xevudy)	Conditional recommendation	• Consider sotrovimab within five days of symptom onset for unvaccinated adults^†^ who do not require supplemental oxygen and have one or more risk factors for disease progression. • Within this group, decisions about the appropriateness of sotrovimab treatment should be based on the individual’s risk of severe disease, including their age, presence of multiple risk factors, and vaccination status (including number of doses and time since last dose or most recent SARS‐CoV‐2 infection). • When infection with Omicron BA.2, BA.4 or BA.5 is confirmed or likely, sotrovimab should only be considered when other treatments are not suitable or available.
	Consensus recommendation	• In addition to unvaccinated adults at risk of progression, also consider sotrovimab within five days of symptom onset for adults who do not require supplemental oxygen and are immunocompromised (regardless of vaccination status), or are at particularly high risk of disease because of advanced age and multiple risk factors. • When infection with Omicron BA.2, BA.4 or BA.5 is confirmed or likely, sotrovimab should only be considered when other treatments are not suitable or available.
Tixagevimab/cilgavimab (Evusheld)	Conditional recommendation	• Consider tixagevimab/cilgavimab within five days of symptom onset for unvaccinated adults^†^ who do not require supplemental oxygen and who have one or more risk factors for disease progression. • Within this group, decisions about the appropriateness of tixagevimab/cilgavimab treatment should be based on the individual’s risk of severe disease, including their age, presence of multiple risk factors, and vaccination status (including number of doses and time since last dose or most recent SARS‐CoV‐2 infection).
	Consensus recommendation	• In addition to unvaccinated adults at risk of progression, also consider tixagevimab/cilgavimab within five days of symptom onset for adults who do not require supplemental oxygen and are immunocompromised, or are at particularly high risk of severe disease because of advanced age and multiple risk factors.

SARS‐CoV‐2 = severe acute respiratory syndrome coronavirus 2.

* Status: 1 August 2022. For full recommendations, see Supporting Information, table 3.

† As the relevant trials excluded people who had received one or more doses of SARS‐CoV‐2 vaccine, the efficacy of these treatment in such people is unclear. See the corresponding consensus recommendation for guidance on use in vaccinated adults or in immunocompromised patients (regardless of vaccination status).

‡ As the relevant trials excluded people who had received one or more doses of SARS‐CoV‐2 vaccine, the efficacy of regdanvimab in such people is unclear. Recommendations for other patient groups are currently under development and will be included in future versions of the guideline.

### Treatments that are not recommended (“do not use”)

The Taskforce has recommended against several treatments and treatment combinations that are either ineffective (neither benefit nor harm the patient) or actively harm the patient (Box 3).

Box 3 “Do not use” and “only in research” recommendations for treatment of people with coronavirus disease 2019 (COVID‐19)^*^

**Table:** 

1. Treatments that should not be used for treating COVID‐19 (current evidence is inadequate)		
Aspirin Azithromycin Colchicine Convalescent plasma (patients requiring oxygen)	Hydroxychloroquine Hydroxychloroquine/azithromycin Interferon β‐1a	Interferon β‐1a/lopinavir/ritonavir Ivermectin Lopinavir/ritonavir
2. Treatments that should only be used to treat COVID‐19 in the context of appropriately controlled randomised clinical trials		
Anakinra Angiotensin 2 receptor agonist C21 Aprepitant Baloxavir marboxil Bamlanivimab Bamlanivimab/etesevimab Bromhexine hydrochloride Camostat mesylate Chloroquine Combined metabolic activators Convalescent plasma (patients not requiring oxygen) Darunavir/cobicistat Doxycycline Dutasteride Enisamium	Favipiravir Fluvoxamine Human umbilical cord mesenchymal stem cells Interferon β‐1a (inhaled) Interferon β‐1b Interferon γ Interferon γ/trefoil factor 2 (IFN‐κ/TFF2) Intravenous immunoglobulin Intravenous immunoglobulin/methylprednisolone Ivermectin/doxycycline Lenzilumab Metformin *N*‐acetylcysteine Nitazoxanide	Peginterferon λ Recombinant human granulocyte colony‐stimulating factor (rHG‐CSF) Regdanvimab Ruxolitinib Sofosbuvir/daclatasvir Sulodexide Telmisartan Tofacitinib Triazavirin Umifenovir Vitamin C Vitamin D analogues Zinc

* Status: 1 August 2022.

The earlier “only in research” recommendation for hydroxychloroquine, based on the unclear findings of seven trials (1081 participants^59^, ^60^, ^61^, ^62^, ^63^, ^64^, ^65^), was revised to “do not use” following publication of the findings of the RECOVERY trial on 15 July 2020, which added data for a further 4716 participants.^66^ Findings from an additional fourteen studies support the conclusion that hydroxychloroquine provides no benefit for patients with COVID‐19 but increases the incidence of adverse events.^13^, ^67^, ^68^, ^69^, ^70^, ^71^, ^72^, ^73^, ^74^, ^75^, ^76^, ^77^, ^78^, ^79^

Recommendations against several other treatments have subsequently been made, primarily on the basis of the findings of the RECOVERY trial: convalescent plasma (fifteen studies, 16 122 participants^80^, ^81^, ^82^, ^83^, ^84^, ^85^, ^86^, ^87^, ^88^, ^89^, ^90^, ^91^, ^92^, ^93^, ^94^), lopinavir/ritonavir (nine studies, 9389 participants^20^, ^68^, ^78^, ^95^, ^96^, ^97^, ^98^, ^99^, ^100^), colchicine (eight studies, 17 782 participants^101^, ^102^, ^103^, ^104^, ^105^, ^106^, ^107^, ^108^), azithromycin (eight studies, 10 728 participants^109^, ^110^, ^111^, ^112^, ^113^, ^114^, ^115^, ^116^), and aspirin (one study, 14 892 participants^117^). The recommendation against interferon β‐1a was primarily based on the findings of the SOLIDARITY trial (four studies, 4646 participants^13^, ^32^, ^118^, ^119^); that against ivermectin was based on the findings of nineteen studies (3869 participants^120^, ^121^, ^122^, ^123^, ^124^, ^125^, ^126^, ^127^, ^128^, ^129^, ^130^, ^131^, ^132^, ^133^, ^134^, ^135^, ^136^, ^137^, ^138^). Recommendations against two dual treatments (hydroxychloroquine/azithromycin, interferon β‐1a/lopinavir/ritonavir) were made because of limited direct evidence for both the absence of a synergistic effect and for the components having little or no effect as stand‐alone treatments.

### Treatments for which there is insufficient evidence of efficacy (“only in research”)

The largest group of recommendations in the Taskforce guideline comprises “only in research” recommendations for treatments for which there is insufficient evidence for determining safety and effectiveness (Box 3). Although preliminary evidence for a beneficial effect in COVID‐19 is available for many of these treatments, further evidence is needed to determine whether the reported findings are reliable indicators of their effectiveness in real‐world practice.

### Chemoprophylaxis

Three treatments have been reviewed for their ability to prevent SARS‐CoV‐2 infection and to improve patient outcomes when used for pre‐ or post‐exposure prophylaxis (Box 4).

Box 4 Recommendations for pre‐ and post‐exposure prophylaxis of coronavirus disease 2019 (COVID‐19)^*^

**Table:** 

Treatment	Category	Recommendation
**Pre‐exposure prophylaxis**		
Hydroxychloroquine	Not recommended	For health care workers without current COVID‐19, do not use hydroxychloroquine for pre‐exposure prophylaxis outside randomised trials with ethics approval.
Tixagevimab/cilgavimab (Evusheld)	Consensus recommendation	Do not routinely use tixagevimab/cilgavimab as pre‐exposure prophylaxis, but it may be considered in exceptional circumstances for people who are severely immunocompromised. Given the limited evidence of benefit or safety, small effect sizes, and absence of evidence for the effectiveness of tixagevimab/cilgavimab for preventing infection by SARS‐CoV‐2 variants of concern, rigorous data collection should be undertaken regarding indications and key outcomes for adults who receive tixagevimab/cilgavimab as pre‐exposure prophylaxis.
**Post‐exposure prophylaxis**		
Casirivimab/imdevimab (Ronapreve)	Conditional recommendation	Consider subcutaneous casirivimab/imdevimab as prophylaxis in seronegative or polymerase chain reaction‐negative close household contacts of people with confirmed SARS‐CoV‐2 infections.
Hydroxychloroquine	Not recommended	For persons exposed to people with SARS‐CoV‐2 infection, do not use hydroxychloroquine for post‐exposure prophylaxis outside randomised trials with ethics approval.
Tixagevimab/cilgavimab (Evusheld)	Only in research settings	For persons exposed to people with SARS‐CoV‐2 infection, do not use tixagevimab/cilgavimab for post‐exposure prophylaxis outside randomised trials with ethics approval.

SARS‐CoV‐2 = severe acute respiratory syndrome coronavirus 2.

* Status: 1 August 2022. For full recommendations, see Supporting Information, table 4.

No benefit for averting laboratory‐confirmed COVID‐19 was found for hydroxychloroquine, used either prior to (three studies, 1884 participants^139^, ^140^, ^141^) or after exposure to people infected with SARS‐CoV‐2 (two studies, 3135 participants^142^, ^143^); its use is therefore not recommended. Casirivimab/imdevimab (Ronapreve) is conditionally recommended for post‐exposure prophylaxis, based on one report of a significant reduction in symptomatic and confirmed infections (one study, 1505 participants^144^). More recently, the Taskforce has given a highly specific consensus recommendation for considering tixagevimab/cilgavimab (Evusheld) for pre‐exposure prophylaxis in people who are severely immunocompromised (one study, 5197 participants^145^).

### Respiratory support

As the major complication of COVID‐19 pneumonia is respiratory deterioration, one of the Taskforce focuses has been developing recommendations regarding the safety and effectiveness of various methods of respiratory support. Unlike the use of therapeutic agents, limited direct and high quality evidence has been available to inform these recommendations.

Based on the best available evidence (primarily systematic reviews and observational data) and expert clinical judgement, the Taskforce Hospital and Acute Care Panel formulated eleven recommendations regarding supplemental oxygen — continuous positive airway pressure and high‐flow nasal oxygen therapy, non‐invasive ventilation, invasive ventilation and extracorporeal membrane oxygenation — and the use of additional therapies, such as prone positioning and recruitment manoeuvres, positive end‐expiratory pressure, and video laryngoscopy. Each of these recommendations includes the caveat that personal protective equipment be used and that these treatments not be provided in shared wards or hospital department cubicles, or during inter‐hospital patient transfer and retrieval (Box 5).

Box 5 Respiratory support recommendations for patients with coronavirus disease 201**9 (COVID‐19)** *

**Table:** 

Topic	Category	Recommendation
Guiding principles of care	Consensus recommendation	• For patients receiving respiratory support, use single and negative pressure rooms when possible; if unavailable, use single rooms or shared ward spaces with cohorting of patients with confirmed COVID‐19. Ensure that precautions to reduce contact, droplet, and airborne transmission are observed. Health care workers should be fully vaccinated and wear fit‐tested N95 masks.
Continuous positive airway pressure (CPAP)	Conditional recommendation	• Consider CPAP for patients with hypoxaemic respiratory failure in whom oxygen saturation is not maintained within target range despite oxygen delivery by nasal prongs or mask. • CPAP therapy is preferred for patients with persistent hypoxaemia associated with COVID‐19 (defined as requiring F_*I*_O_2_ ≥ 0.4 to maintain oxygen saturation in target range). Adjust continuous positive airway pressure as required; most patients require pressures of 10–12 cmH_2_O. Excessive pressure may increase risk of pneumothorax. Titrate oxygen to maintain saturation in the target range. Direct evidence for the value of bi‐level positive pressure support is currently insufficient. • If CPAP is not available or not tolerated, consider high‐flow nasal oxygen (HFNO), with the same safety parameters. • Monitor patients receiving CPAP or HFNO closely at all times; liaise with intensive care unit in case of deterioration. Do not delay endotracheal intubation and invasive mechanical ventilation of a patient whose condition deteriorates despite optimised, less invasive respiratory therapies.
Respiratory management of patients whose condition deteriorates	Consensus recommendation	• Do not delay endotracheal intubation and mechanical ventilation in a patient whose condition deteriorates despite optimised, less invasive respiratory therapies.
Video laryngoscopy	Conditional recommendation	• In adults undergoing endotracheal intubation, prefer video laryngoscopy to direct laryngoscopy if trained operator is available.
Neuromuscular blockers	Conditional recommendation against	• For mechanically ventilated adults and moderate to severe acute respiratory distress syndrome, do not routinely use continuous infusions of neuromuscular blocking agents.
Positive end‐expiratory pressure (PEEP)	Consensus recommendation	• For mechanically ventilated adults and moderate to severe acute respiratory distress syndrome, prefer higher PEEP strategy (PEEP > 10 cmH_2_O) to lower PEEP strategy. • We do not expect to update this low priority recommendation in the near future, but will continue to review the published evidence.
Prone positioning	Consensus recommendation	• For mechanically ventilated adults with hypoxaemia despite optimised ventilation, consider prone positioning for more than 12 hours a day.
	Conditional recommendation	• For adults with respiratory symptoms receiving any form of supplemental oxygen therapy and not yet intubated, consider prone positioning for at least three hours a day, if tolerated, and closely monitor the patient. Prone positioning should not delay endotracheal intubation and mechanical ventilation in a patient whose condition deteriorates despite optimised less invasive respiratory therapies.
Prone positioning and cardiopulmonary resuscitation (CPR)	Consensus recommendation	• For patients in prone position who require CPR, return the patient to supine position and commence resuscitation, when safe and feasible. • If returning the patient to supine position is not safe and feasible, commence CPR in prone position. Once it is safe and feasible, return the patient to supine position and continue CPR.
Recruitment manoeuvres	Consensus recommendation	• For mechanically ventilated adults with hypoxaemia despite optimised ventilation, consider recruitment manoeuvres, but not staircase or stepwise (incremental PEEP) recruitment manoeuvres.
Extracorporeal membrane oxygenation (ECMO)	Conditional recommendation	• Consider early referral to an ECMO centre for mechanically ventilated adults who develop refractory respiratory failure despite optimised ventilation, including prone positioning and neuromuscular blockers.

F_*I*_O_2_ = fraction of inspired oxygen.

* Status: 1 August 2022. For full recommendations, see Supporting Information, table 5.

### Supportive recommendations

The Taskforce developed several supportive care recommendations. One focus has been the use of anticoagulants for venous thromboembolism prophylaxis in patients with COVID‐19. The REMAP‐CAP trial^146^ found that therapeutic dose anticoagulation achieved a statistically significant greater reduction in blood clots than prophylactic dosing, but increased the risk of significant bleeding. Consequently, the Taskforce conditionally recommended against routinely offering therapeutic anticoagulation, instead supporting prophylactic dose anticoagulation in patients with moderate, severe, or critical COVID‐19.

Another important consideration is whether to maintain or cease therapies for other diseases in patients with COVID‐19. A systematic review of observational studies found no adverse effects of continued angiotensin‐converting enzyme inhibitor or angiotensin II receptor blocker therapy in patients with COVID‐19,^147^ and the Taskforce recommended these treatments be maintained. A further consensus recommendation supports maintaining steroid therapy for people with asthma or chronic obstructive pulmonary disease. Three linked consensus recommendations advise cessation of oral menopausal hormone therapy in women with severe or critical COVID‐19, and consideration of cessation for women with mild or moderate COVID‐19, because of the greater risk of venous thromboembolism in these patients.

Finally, the Taskforce has reviewed a large multicentre study of the timing of surgery after COVID‐19.^148^ Given the reported increase in harms, the Taskforce conditionally recommended against elective surgery within eight weeks of recovery from acute COVID‐19, and conditionally recommended multisystem pre‐operative assessment of people who subsequently undergo surgery (Box 6).

Box 6 Additional supportive recommendations for managing adults with coronavirus disease 2019 (COVID‐19)*

**Table:** 

Treatment	Category	Recommendation
**Venous thrombo‐embolism prophylaxis**	Conditional recommendation	• Use prophylactic anticoagulant doses, preferably low molecular weight heparin (LMWH) (eg, enoxaparin 40 mg once daily or dalteparin 5000 IU once daily), in adults with moderate, severe, or critical COVID‐19 unless contraindicated (eg, risk of major bleeding). If the estimated glomerular filtration rate is below 30 mL/min/1.73 m^2^, unfractionated heparin or clearance‐adjusted LMWH doses may be used (eg, enoxaparin 20 mg once daily).
	Conditional recommendation against	• Do not routinely offer therapeutic anticoagulant doses to adults with moderate, severe, or critical COVID‐19. There is no additional indication for therapeutic anticoagulant dosing for adults with severe or critical COVID‐19 beyond current standard best practice.
**Therapies for comorbid conditions**		
ACEIs/ARBs (hypertension)	Recommended	• In patients receiving ACEIs/ARBs, no evidence supports deviating from usual care; these medications should be continued unless contraindicated.
Steroids (asthma, COPD)	Consensus recommendation	• Use inhaled or oral steroids for managing co‐existing asthma or COPD as usual for viral exacerbation of asthma or COPD. Do not use nebulisers.
Oestrogen‐containing therapies	Consensus recommendation	• In women taking oral menopausal hormone therapy (MHT), manage these medications as usual. In women who stop or suspend oral MHT, review the indication for doing so and consider transitioning to a transdermal preparation. Manage transdermal MHT as usual.
	Consensus recommendation	• In women using oestrogen‐containing contraception, manage these medications as usual. • In women who stop or suspend contraception while they have COVID‐19, restart contraception at the time of discharge or when acute symptoms have resolved.
**Surgery following COVID‐19 infection**	Conditional recommendation against	• Do not routinely perform elective surgery within seven weeks of recovery from acute illness or following confirmed SARS‐CoV‐2 infection unless the risk of deferring surgery is considerable, such as disease progression or clinical priority. Very low risk or low risk procedures, such as endoscopy or skin incision, should be considered if warranted by clinical need.
	Conditional recommendation	• For people undergoing elective surgery following confirmed SARS‐CoV‐2 infection, consider a multisystem pre‐operative assessment in consultation with a unit familiar with the assessment of people recovering from COVID‐19.

ACEI = angiotensin‐converting enzyme inhibitor; ARB = angiotensin II receptor blocker; COPD = chronic obstructive pulmonary disease; SARS‐CoV‐2 = severe acute respiratory syndrome coronavirus 2.

* Status: 1 August 2022. For full recommendations, see Supporting Information, table 6.

## Discussion

From its inception in March 2020, the National COVID‐19 Clinical Evidence Taskforce has implemented a robust process to continually maintain up‐to‐date recommendations for treating people with COVID‐19. It involves daily searches for published evidence, rapid appraisal and analysis of study findings, and frequent meetings of clinical expert panels in which recommendations are developed and ratified. During its first two years, the guideline was updated 106 times, and its scope has increased from nine to 176 recommendations covering therapeutic treatment and respiratory support. The complete current version of the guideline, including specific recommendations for particular patient groups, clinical flowcharts, and decision support tools, is available at https://covid19evidence.net.au.

The work of the Taskforce will continue to evolve, particularly in areas such as the post‐COVID‐19 syndrome (“long COVID”), for which a treatment and care evidence base remains to be established. In addition, factors such as the vaccination status of trial participants, the paucity of direct evidence for the treatment of people infected with more recent SARS‐CoV‐2 variants, and laboratory findings regarding the activity of monoclonal antibodies against such variants, will be considered.

The ability to capture and assess new evidence quickly, facilitated by the committed work of the more than 250 volunteer members of the guideline panels, leadership group, steering committee, and other stakeholders, has shown that it is possible to maintain clear, up‐to‐date guidelines for a high priority area in which evidence is rapidly evolving, while speaking with a unified voice.

## Open access

Open access publishing facilitated by Monash University, as part of the Wiley–Monash University agreement via the Council of Australian University Librarians.

## Competing interests

No relevant disclosures.

## Provenance

Not commissioned; externally peer reviewed.

## Supporting information


Appendix S1.
