# Supplementary material for: Care for adults with COVID‐19: living guidelines from the National COVID‐19 Clinical Evidence Taskforce
Source: Med J Aust. 2022 Sep 23;217(7):368–78. doi: 10.5694/mja2.51718 (PMC9538623; doi:10.5694/mja2.51718)
Supplement: Supplementary file 1 — Appendix S1. [file MJA2-217-368-s001.pdf]

The Medical Journal of Australia

Supporting Information

Full guideline (version 61.0)

This appendix was part of the submitted manuscript and has been peer reviewed.

It is posted as supplied by the authors.

Appendix to: White H, McDonald SJ, Barber B, et al. Care for adults with COVID-19: living guidelines from the National COVID-19 Clinical Evidence Taskforce. *Med J Aust* 2022; doi: 10.5694/mja2.51718.

The recommendations below were taken from version 61.0 of the Australian guidelines for the clinical care of people with COVID-19, published within MAGICApp by the National COVID-19 Clinical Evidence Taskforce on 1 August 2022. As the guideline is constantly evolving based on new evidence and information, the recommendations may have changed since publication. For the current version of each recommendation, please visit MAGICApp (https://app.magicapp.org/#/guideline/6202).

Table 1. Definitions of disease severity for adults with coronavirus disease 2019 (COVID-19) (guideline section 4.1)

Severity	Definition
**Mild illness**	An individual with no clinical features suggestive of moderate or more severe disease:<br>• no OR mild symptoms and signs (fever, cough, sore throat, malaise, headache, muscle pain, nausea, vomiting, diarrhoea, loss of taste and smell)<br>• no new shortness of breath or difficulty breathing on exertion<br>• no evidence of lower respiratory tract disease during clinical assessment or on imaging (if performed)
**Moderate illness**	A stable patient with evidence of lower respiratory tract disease:<br>• during clinical assessment, such as<br>○ oxygen saturation 92–94% on room air at rest<br>○ desaturation or breathlessness with mild exertion<br>• or on imaging
**Severe illness**	A patient with signs of moderate disease who is deteriorating<br>OR<br>A patient meeting any of the following criteria:<br>• respiratory rate ≥ 30 breaths/min<br>• oxygen saturation < 92% on room air at rest or requiring oxygen<br>• lung infiltrates > 50%
**Critical illness**	A patient meeting any of the following criteria:<br>• Respiratory failure (defined as any of)<br>○ severe respiratory failure ($PaO_2/FiO_2 < 200$)<br>○ respiratory distress or acute respiratory distress syndrome (ARDS)<br>○ deteriorating despite non-invasive forms of respiratory support (i.e. non-invasive ventilation [NIV], or high-flow nasal oxygen [HFNO])<br>○ requiring mechanical ventilation<br>• hypotension or shock<br>• impairment of consciousness<br>• other organ failure

PaO_2/FiO_2 – ratio of arterial oxygen partial pressure to fractional inspired oxygen

Table 2. Recommended drug treatments for patients with COVID-19 who require oxygen (guideline section 6.1)

**Casirivimab plus imdevimab (Ronapreve™)**	
**Conditional recommendation**	**Consider using casirivimab plus imdevimab in seronegative adults hospitalised with moderate-to-critical COVID-19**<br><br>**Where infection with Omicron BA.1, BA.2, BA.4 or BA.5 is confirmed or considered likely, use of casirivimab plus imdevimab should only be considered where other treatments are not suitable or available.**
Remark	*While the clinical evidence supports use of casirivimab plus imdevimab to treat mild COVID-19, there is no clinical evidence to evaluate its effectiveness against the Omicron variant or BA.1, BA.2, BA.4 or BA.5 sub-variants. The Taskforce is aware of* in vitro *data that suggest potentially reduced efficacy against these variants and while the clinical implications of this are not certain, given the availability of other treatments, where infection with Omicron BA.2, BA.4 or BA.5 is confirmed or considered likely, use of casirivimab plus imdevimab should not be considered unless other treatments are unsuitable or unavailable.*<br><br>In patients hospitalised with moderate-to-critical COVID-19 who are seronegative (no detectable SARS-CoV-2 antibodies), casirivimab plus imdevimab probably reduces the risk of death. Because of this, the Taskforce gives a conditional recommendation for casirivimab plus imdevimab both within and outside the context of a randomised trial.<br><br>The Taskforce notes that the RECOVERY trial administered a single intravenous 8000 mg dose of REGEN-COV (4000 mg casirivimab plus 4000 mg imdevimab in 250 ml 0.9% saline) and assessed baseline serostatus using the Oxford (anti-spike IgG) immunoassay[1], the use of which has been supported by the UK National SARS-CoV-2-Serology Assay Evaluation Group.[2]<br><br>It should be noted that the study by Somersan-Karakaya initially included patients requiring ventilation but ceased their recruitment due to safety concerns, however no safety data were provided for these patients.[3]<br><br>The Taskforce is aware of concerns about the potential for decreased effectiveness of casirivimab plus imdevimab against the Omicron variant, based on *in vitro* data. We will update this recommendation as definitive evidence becomes available.<br><br>The sponsor has not submitted an application to the Therapeutics Goods Administration for use of casirivimab plus imdevimab in hospitalised patients and it is presently not approved for this indication.<br><br>*This is a <u>moderate priority</u> recommendation and will be updated when new evidence becomes available that is likely to impact the direction or strength of the recommendation.*

**Corticosteroids (systemic)**	
**Recommended**	**Use intravenous or oral dexamethasone for up to 10 days (or acceptable alternative regimen) in adults with COVID-19 who require oxygen (including mechanically ventilated patients).**
Remark	The suggested regimen of corticosteroid use is 6 mg of dexamethasone (oral or intravenous) daily for up to 10 days. In patients for whom dexamethasone is not available, acceptable alternative regimens include:<br>• hydrocortisone: intravenous (50 mg), every 6 hours for up to 10 days<br>• prednisolone: oral (50 mg), daily for up to 10 days<br>• methylprednisolone may also be an acceptable alternative, however the most appropriate dosage is uncertain<br>It is unclear whether older people living with frailty or cognitive impairment, or those requiring palliative care were included in the studies on which this recommendation is based. Until further evidence in these populations is available, the Taskforce does not believe a different recommendation should apply, unless contraindicated.<br><br>*This is a <u>moderate priority</u> recommendation and will be updated when new evidence becomes available that is likely to impact the direction or strength of the recommendation.*
**Conditional recommendation against** ***Remark***	**Do not routinely use dexamethasone (or other systemic corticosteroid) to treat COVID-19 in adults who do not require oxygen**.<br><br>Corticosteroids may still be considered for other evidence-based indications in people who have COVID-19.<br><br>*This is a <u>moderate priority</u> recommendation and will be updated when new evidence becomes available that is likely to impact the direction or strength of the recommendation.*
**Baricitinib**	
**Conditional recommendation** ***Remark***	**Consider using baricitinib in adults hospitalised with COVID-19 who require supplemental oxygen.**<br><br>In patients hospitalised with COVID-19 who require supplemental oxygen, baricitinib probably reduces the risk of death. Because of this, the Taskforce gives a conditional recommendation for baricitinib use both within and outside the context of a randomised trial.<br><br>In accordance with the RECOVERY, ACTT-2 and COV-BARRIER studies[4-6], baricitinib should be administered as a 4 mg oral daily dose for up to 14 days. In patients receiving more intensive oxygen delivery where oral administration is not feasible, administer via nasogastric tube. Consider using a reduced dose of 2 mg daily in patients with an eGFR of between 30 and 60 mL/min/1.73m^2.<br><br>The Taskforce previously recommended baricitinib for use in patients who required supplemental oxygen but not mechanical ventilation or ECMO due to the absence of direct evidence within this population. Data from the COV-BARRIER extension study suggests baricitinib is safe and effective in patients hospitalised with COVID-19 who require mechanical ventilation or ECMO. The Taskforce has subsequently revised the recommendation to include these patients.

	The Taskforce notes the current **critical shortage of tocilizumab**. Therefore, in patients who are receiving supplemental oxygen, baricitinib should be considered as an alternative to tocilizumab, unless contraindicated. See the statement from the TGA and Medicine Availability Working Group regarding the shortage.[7]<br><br>The RECOVERY trial demonstrated a benefit when using baricitinib in conjunction with corticosteroids, tocilizumab or remdesivir[4], however the Taskforce notes that the concomitant use of two or more immunomodulatory agents may increase the risk of side effects such as opportunistic infection.<br><br>*This is a <u>moderate priority</u> recommendation and will be updated when new evidence becomes available that is likely to impact the direction or strength of the recommendation.*
**Sarilumab**	
**Conditional recommendation**<br><br>***<u>Remark</u>***	**Consider using sarilumab for the treatment of COVID-19 in adults who require high-flow oxygen, non-invasive ventilation or invasive mechanical ventilation.**<br><br>In patients hospitalised with COVID-19 who require high-flow oxygen, non-invasive ventilation or invasive mechanical ventilation, sarilumab probably reduces the risk of death. Because of this, the Taskforce gives a conditional recommendation for sarilumab use both within and outside the context of a randomised trial.<br><br>Uncertainty remains whether sarilumab impacts risk of death in patients who require no ventilatory support or low-flow oxygen.<br><br>*This is a <u>moderate priority</u> recommendation and will be updated when new evidence becomes available that is likely to impact the direction or strength of the recommendation.*
**Tocilizumab**	
**Conditional recommendation**<br><br>***<u>Remark</u>***	**Consider using tocilizumab for the treatment of COVID-19 in adults who require supplemental oxygen, particularly where there is evidence of systemic inflammation.**<br><br>In patients hospitalised with COVID-19 who require supplemental oxygen, tocilizumab probably reduces the risk of death. Because of this, the Taskforce gives a conditional recommendation for tocilizumab use both within and outside the context of a randomised trial unless contraindicated (e.g. patients with other active, severe infections).<br><br>The Taskforce notes the current **critical shortage of tocilizumab**. Therefore, in patients who are receiving supplemental oxygen, baricitinib should be considered as an alternative to tocilizumab, unless contraindicated. For the TGA and Medicine Availability Working Group statements regarding the shortage.[7]<br>In accordance with the RECOVERY trial, tocilizumab should be administered as a single intravenous infusion over 60 minutes.[8] The suggested dose is dependent on body weight:

	• Patients > 90 kg: 800 mg tocilizumab<br>• Patients 66–90 kg: 600 mg tocilizumab<br>• Patients 41–65 kg: 400 mg tocilizumab<br>• Patients ≤ 40 kg: 8 mg/kg tocilizumab<br><br>In the RECOVERY trial, 29% of patients received a second dose 12–24 hours after the first dose, although results were not reported separately for this population. The decision to administer a second dose of tocilizumab should take into consideration its availability.<br><br>Both RECOVERY and REMAP-CAP trials demonstrated a significant benefit when using corticosteroids in conjunction with tocilizumab[4,9]. Use of combined tocilizumab and corticosteroids should be considered in patients hospitalised with COVID-19 who require oxygen, however the optimal sequencing of tocilizumab and corticosteroid use is unclear. In addition, the RECOVERY trial demonstrated a benefit when using tocilizumab in conjunction with baricitinib[4], however there are limited data available to evaluate the safety of this combination.<br><br>As tocilizumab inhibits the production of C-reactive protein (CRP), a reduction in CRP should not be used as a marker of clinical improvement.<br><br>*This is a <u>high priority</u> recommendation and will be updated as soon as new evidence becomes available.*
**Remdesivir**	
**Conditional recommendation**<br><br>***<u>Remark</u>***	**Consider using remdesivir in adults with COVID-19 who require oxygen but do not require non-invasive or invasive ventilation.**<br><br>In patients hospitalised with COVID-19 who do not require ventilation (invasive or non-invasive mechanical ventilation or extracorporeal membrane oxygenation [ECMO]) remdesivir probably reduces the risk of death. Because of this, the Taskforce gives a conditional recommendation for remdesivir use both within and outside the context of a randomised trial.<br><br>It is unclear whether older people or those requiring palliative care were included in the studies on which this recommendation is based. Until further evidence in these populations is available, the Taskforce does not believe a different recommendation should apply, unless contraindicated.<br><br>We are aware of the difference between our recommendations for remdesivir and those currently issued by the World Health Organization.[10] For a full description of the rationale underpinning this decision please see here.[11]<br><br>It is unclear which regimen of remdesivir (5-day or 10-day) provides the optimal duration of treatment. In Australia, criteria for accessing remdesivir from the National Medical Stockpile limits the treatment course to 5 days for eligible patients.<br><br>*This is a <u>high priority</u> recommendation and will be updated as soon as new evidence becomes available.*

SARS-CoV-2 – Severe acute respiratory syndrome coronavirus 2; ECMO – extracorporeal membrane oxygenation; TGA – Therapeutic Goods Administration

Table 3. Drug treatments for individuals with COVID-19 who do not require oxygen (guideline section 6.1)

**Casirivimab plus imdevimab**	
**Conditional recommendation**	**Consider using casirivimab plus imdevimab within 7 days of symptom onset in adults with COVID-19 who do not require oxygen and who have one or more risk factors for disease progression**.<br><br>Where infection with Omicron BA.1, BA.2, BA.4 or BA.5 is confirmed or considered likely, use of casirivimab plus imdevimab should only be considered where other treatments are not suitable or available.
Remark	*While the clinical evidence supports use of casirivimab plus imdevimab to treat mild COVID-19, there is no clinical evidence to evaluate its effectiveness against the Omicron variant or BA.1, BA.2, BA.4 or BA.5 sub-variants. The Taskforce is aware of in vitro data that suggest potentially reduced efficacy against these variants and while the clinical implications of this are not certain, given the availability of other treatments, where infection with Omicron BA.2, BA.4 or BA.5 is confirmed or considered likely, use of casirivimab plus imdevimab should not be considered unless other treatments are unsuitable or unavailable.*<br><br>*The Taskforce is aware of concerns about the potential for decreased effectiveness of casirivimab plus imdevimab against the Omicron variant, based on in vitro data.[12] In contexts where Omicron is the dominant variant, do not routinely use casirivimab plus imdevimab for the treatment of COVID-19, unless there is reason to believe the patient has another variant.*<br><br>In adult outpatients with mild COVID-19, casirivimab plus imdevimab probably reduces hospitalisation and incidence of adverse events. Because of this, the Taskforce gives a conditional recommendation for casirivimab plus imdevimab both within and outside the context of a randomised trial.<br><br>Included data comes from the three-phase REGEN-COV trial[13,14] in which patients with one or more risk factors for disease progression received either 1200 mg, 2400 mg or 8000 mg casirivimab plus imdevimab. Based on inclusion criteria of the trial, risk factors for disease progression include:<br><br>• Age ≥ 50 years<br>• Obesity (BMI ≥ 30 kg/m2)<br>• Cardiovascular disease (including hypertension)<br>• Chronic lung disease (including asthma)<br>• Type 1 or 2 diabetes mellitus<br>• Chronic kidney disease, including those that are on dialysis<br>• Chronic liver disease<br>• Immunocompromise (including in individuals with rheumatoid arthritis, HIV/AIDS and systemic lupus erythematosus receiving immunosuppressive treatment)

	As there is insufficient data supporting one dose over another, the Taskforce recommends that the most frequently used dose across the studies (1200 mg) should be administered within 7 days of onset of symptoms.<br><br>The efficacy of casirivimab plus imdevimab in vaccinated or immunocompromised patients with mild or asymptomatic COVID-19 is not known.<br><br>As of 7 March 2022, the Taskforce has made conditional recommendations supporting the use of sotrovimab, casirivimab plus imdevimab, nirmatrelvir plus ritonavir, remdesivir and molnupiravir in adult outpatients with mild COVID-19. The relative effectiveness of these agents, or the effectiveness of these agents when used in combination, is unclear.<br><br>*This is a <u>moderate priority</u> recommendation and will be updated when new evidence becomes available that is likely to impact the direction or strength of the recommendation.*
**Corticosteroids (inhaled)**	
**Conditional recommendation**<br><br>***<u>Remark</u>***	**Consider using inhaled corticosteroids (budesonide or ciclesonide) within 14 days of symptom onset in adults with COVID-19 who do not require oxygen and who have one or more risk factors for disease progression.**<br><br>In patients with confirmed COVID-19 who do not require oxygen, inhaled corticosteroids probably reduce hospitalisation. In individuals who are subsequently hospitalised due to disease progression, inhaled corticosteroids probably decrease the requirement for supplemental oxygen if taken within 14 days of onset of symptoms.<br><br>Five trials provide evidence for inhaled corticosteroids in the treatment of COVID-19, three comparing ciclesonide with standard care, and two comparing budesonide with standard care or placebo. Results are primarily based on the PRINCIPLE trial[15] in which adults were treated with inhaled budesonide (by breath-actuated inhaler) 800 µg twice daily for up to 14 days. Based on the inclusion criteria for this trial, risk factors for disease progression include age ≥ 65 years or ≥ 50 years with one or more of the following comorbidities:<br><br>• Diabetes (not treated with insulin)<br>• Heart disease and/or hypertension<br>• Asthma or lung disease<br>• Weakened immune system due to a serious illness or medication (e.g. chemotherapy)<br>• Mild hepatic impairment<br>• Stroke or other neurological problem<br><br>Approximately 11% and 1% of participants had received one or two doses of vaccine at enrolment, respectively, however results were not reported separately for this population.<br><br>Budesonide and ciclesonide are safe to use in pregnant and breastfeeding women.

	This is a moderate priority recommendation and will be updated when new evidence becomes available that is likely to impact the direction or strength of the recommendation.
**Molnupiravir (Lagevrio™)**	
**Consensus recommendation**	**Consider using molnupiravir within 5 days of symptom onset in unvaccinated* adults with COVID-19 who do not require oxygen and who have one or more risk factors for disease progression, where other treatments (such as remdesivir or nirmatrelvir plus ritonavir) are not suitable or available.**<br><br>**Within the patient population for which molnupiravir is recommended for use (see Remark), decisions about the appropriateness of treatment with molnupiravir should be based on the individual's risk of severe disease, including their age, presence of multiple risk factors, and vaccination status (including number of doses and time since last dose/ or timing of most recent infection).**
Remark	Based on available data, molnupiravir may reduce hospitalisation or death in individuals with PCR-confirmed COVID-19 and mild illness when treated within 5 days of onset of symptoms, however the evidence is limited, effect sizes are small and there are limited safety data. Until further evidence is available, use of molnupiravir should only be considered where other treatments (such as remdesivir or nirmatrelvir plus ritonavir) are not suitable or available.<br><br>The Taskforce notes the high level of efficacy in reduction of the composite outcome of hospitalisation or death observed within the interim analysis (n=762; 68 fewer per 1000) and the subsequent reduction in efficacy within the final analysis (n=1408; 29 fewer per 1000)[16]. It is unclear whether or to what extent the significant proportional increase in patients infected with the Delta variant between the interim and final analyses contributes to the observed reduction in efficacy. The efficacy of molnupiravir against the Omicron variant is not known.<br><br>Results are based on a single trial in which unvaccinated adults were treated with 800 mg of molnupiravir twice daily for 5 days. Based on the inclusion criteria for this trial, risk factors for disease progression include the following:<br><br>• Age ≥ 60 years<br>• Obesity (BMI ≥ 30 kg/m^2)<br>• Chronic kidney disease (eGFR 30–60 mL/min/1.73m^2 by MDRD), excluding patients on dialysis<br>• Serious heart conditions such as heart failure, coronary artery disease or cardiomyopathies<br>• Chronic obstructive pulmonary disease<br>• Active cancer (excluding minor cancers not associated with immunosuppression, e.g. basal cell carcinomas)<br>• Immunocompromised state following solid organ transplant<br>• Sickle cell disease<br>• Diabetes mellitus

	Pregnant & breastfeeding women and children & adolescents were not included in the trial. There are no clinical data for the use of molnupiravir in pregnant women, however animal reproductive studies indicate that molnupiravir may have embryolethal and teratogenic effects at high doses and may result in reduced fetal growth.<br><br>Contraception is recommended until 4 days after the final dose of molnupiravir in sexually active women of childbearing potential, and for 3 months in men who are sexually active with a partner of childbearing potential (TGA PI).[17]<br><br>The Taskforce notes the preprint publication of a trial of 1220 non-hospitalised adults with mild COVID-19 and at least one risk factor for disease progression (24 February 2022 in SSRN[18]). No patients met the primary endpoint of hospital admission for > 24 hours requiring oxygen supplementation. Molnupiravir did reduce the median time to clinical improvement (10 days vs 14 days). This study is currently under review and an updated recommendation will be included in a future version of the guideline.<br><br>The efficacy of molnupiravir in vaccinated or immunocompromised patients is unknown.<br><br>In the absence of reliable evidence, the Taskforce has developed this recommendation based on their clinical expertise and experience.<br><br>Molnupiravir is currently listed within the Australian Pharmaceutical Benefits Scheme.[19]<br><br>*This is a <u>high priority</u> recommendation and will be updated as soon as new evidence becomes available.*
**Consensus recommendation**<br><br><br><br><br>***<u>Remark</u>***	**In addition to at-risk unvaccinated adults, also consider using molnupiravir within 5 days of symptom onset in adults with COVID-19 who do not require oxygen and are immunocompromised; or are at particularly high risk of severe disease on the basis of advanced age and multiple risk factors; AND where other treatments (such as remdesivir or nirmatrelvir plus ritonavir) are not suitable or available.**<br><br>Decisions about the appropriateness of molnupiravir should be based on the individual’s risk of severe disease, including their age, presence of multiple risk factors, and vaccination status (including number of doses and time since last dose/ or timing of most recent infection).<br><br>Available research does not currently provide enough evidence to determine the benefits of molnupiravir in specific subgroups of patients. In the absence of definitive evidence, the Taskforce has arrived at a consensus recommendation based on their combined clinical expertise to guide clinical decisions about which patients are most likely to benefit from molnupiravir.<br><br>There is no evidence evaluating the effectiveness of molnupiravir in individuals who have received any COVID-19 vaccine. Given this, and the lower risk of deterioration in these people, it is unlikely that molnupiravir will have a significant treatment benefit in individuals who have received a vaccine dose or had a SARS-CoV-2 infection in the past 6 months, unless the patient is immunocompromised.

	There is limited evidence on the effectiveness of molnupiravir in immunosuppressed patients. However, given the likely higher risk of deterioration in these patients, and the absence of reasons to believe otherwise, it is likely that molnupiravir will be beneficial for immunocompromised patients.<br><br>Immunocompromising conditions include:<br><br>• Primary or acquired immunodeficiency<br>○ Haematologic neoplasms: leukaemias, lymphomas, myelodysplastic syndromes<br>○ Post-transplant: solid organ (on immunosuppressive therapy), haematopoietic stem cell transplant (within 24 months)<br>○ Immunocompromised due to primary or acquired (HIV/AIDS) immunodeficiency<br>○ Other significantly immunocompromising conditions<br>• Immunosuppressive therapy (current or recent)<br>○ Chemotherapy, whole body radiotherapy or total lymphoid irradiation<br>○ High-dose corticosteroids (≥ 20 mg of prednisone per day, or equivalent) for ≥ 14 days<br>○ All biological disease-modifying anti-rheumatic drugs (DMARDs) and most other (e.g. conventional synthetic) DMARDs[20]<br><br>In the absence of reliable evidence, the Taskforce has developed this recommendation based on their clinical expertise and experience.
**Nirmatrelvir plus ritonavir (Paxlovid™)**	
**Conditional recommendation**	**Consider using nirmatrelvir plus ritonavir (Paxlovid) within 5 days of symptom onset in unvaccinated adults* with COVID-19 who do not require oxygen and who have one or more risk factors for disease progression.**<br><br>**Within the patient population for which nirmatrelvir plus ritonavir is conditionally recommended for use (see Remark), decisions about the appropriateness of treatment with nirmatrelvir plus ritonavir should be based on the individual's risk of severe disease, including their age, presence of multiple risk factors, and vaccination status (including number of doses and time since last dose/ or timing of most recent infection).**<br><br>**** Individuals who had received one or more doses of SARS-CoV-2 vaccine were excluded from the trial. The efficacy of nirmatrelvir plus ritonavir is unclear in individuals who have received any COVID-19 vaccine. See consensus recommendation for guidance on use of nirmatrelvir plus ritonavir in vaccinated adults or in immunocompromised patients regardless of vaccination status.***
Remark	In adults with confirmed COVID-19 who do not require oxygen, nirmatrelvir plus ritonavir (Paxlovid) probably decreases the risk of hospitalisation if taken within 5 days of onset of symptoms.<br><br>Results are based on a single phase 2/3 trial comparing nirmatrelvir plus ritonavir with placebo in 2246 unvaccinated adults with PCR-confirmed COVID-19 and mild illness (EPIC-HR).[21] Within this trial participants were treated with oral nirmatrelvir/ritonavir 300 mg/100 mg twice daily for 5 days.

	The benefit of nirmatrelvir plus ritonavir is likely to be greatest in those with the greatest risk of severe disease. Based on the population included within the trial, evidence demonstrates a reduction in hospitalisation when used in individuals with one or more of the following risk factors for disease progression:<br><br>• Age ≥ 60 years<br>• Diabetes (requiring medication)<br>• BMI ≥ 25 kg/m2<br>• Cardiovascular disease<br>• Hypertension<br>• Chronic lung disease<br><br>There were insufficient numbers of participants with the following risk factors to determine the extent to which nirmatrelvir plus ritonavir impacts hospitalisation or death, however as these conditions frequently result in poorer outcomes for patients following SARS-CoV-2 infection, they will likely benefit from treatment:<br><br>• Chronic kidney disease (but where the eGFR ≥ 30 mL/min)*<br>• Immunosuppressed (e.g. bone marrow or organ transplantation, primary immune deficiencies, prolonged use of immune-weakening medications)<br>• Medical related technological dependence (e.g. CPAP not related to COVID-19)<br>• HIV positive (viral load < 400 copies/mL)<br>• Neurodevelopmental disorders (e.g. cerebral palsy, Down syndrome)<br>• Cancer (other than localised skin cancer)<br>• Sickle cell disease<br><br>** Individuals with an eGFR < 30 mL/min were excluded from the trial. In individuals with CKD and an eGFR of 30–60 mL/min, the dose of nirmatrelvir should be halved; i.e. nirmatrelvir/ritonavir 150/100 mg twice daily for 5 days (FDA EUA[22]).*<br><br>Pregnant and breastfeeding women and children and adolescents were not included in the trial.<br><br>Individuals who had received one or more doses of SARS-CoV-2 vaccine were excluded from the trial.<br><br>The study was conducted before the Omicron variant was prevalent. As a result, there are no data regarding the effectiveness of nirmatrelvir plus ritonavir specific to the Omicron variant.<br><br>The Taskforce notes the interim results of the EPIC-SR study from Pfizer. This trial is evaluating nirmatrelvir plus ritonavir (Paxlovid) in unvaccinated adults at low risk of hospitalisation or death, or vaccinated adults with one or more risk factors for progressing to severe

	illness. An analysis at 80% of enrolled patients found that 0.7% (3/428) of those who received Paxlovid were hospitalised compared with 2.4% (10/426) of patients who received placebo. No deaths were reported. These data will be reviewed when the results become available.<br><br>Ritonavir is an inhibitor, inducer and substrate of many enzymes and transporters involved in drug disposition and metabolism. It is a strong inhibitor of CYP3A, reducing the hepatic metabolism and increasing the concentration of nirmatrelvir and other CYP3A substrates. Coadministration of nirmatrelvir-ritonavir is contraindicated with drugs that are highly dependent upon CYP3A for clearance where an elevated concentration may be dangerous (e.g. anti-arrhythmics, antipsychotics, statins, anti-inflammatories, anti-cancer drugs and anticoagulants). Coadministration is also contraindicated with potent CYP3A inducers (e.g. anti-epileptics, rifampin, St John's wort), which can reduce concentrations of nirmatrelvir and/or ritonavir, reducing efficacy and increasing resistance. Induction persists many days after cessation due to time required for clearance of the inducing drug and of the induced CYP3A.<br><br>Evidence demonstrates a likely weak interaction between nirmatrelvir-ritonavir and budesonide, resulting in increased budesonide concentrations. There are no other expected interactions between nirmatrelvir-ritonavir and other therapeutics currently recommended for the treatment of COVID-19 within the Taskforce guidelines. It is crucial that consideration is given to the potential for complex, serious drug-drug interactions when prescribing and administering nirmatrelvir plus ritonavir with other medications (see Liverpool Interaction Checker[23] and TGA PI[24]).<br>As of 07 March 2022, the Taskforce has made conditional recommendations supporting the use of sotrovimab, casirivimab plus imdevimab, nirmatrelvir plus ritonavir, remdesivir and molnupiravir in adult outpatients with mild COVID-19. The relative effectiveness of these agents, or the effectiveness of these agents when used in combination, is unclear.<br><br>Nirmatrelvir plus ritonavir is currently listed within the Australian Pharmaceutical Benefits Scheme.[25]<br><br>*This is a <u>high priority</u> recommendation and will be updated as soon as new evidence becomes available.*
**Consensus recommendation**<br><br><br><br>***<u>Remark</u>***	**In addition to at-risk unvaccinated adults, also consider using nirmatrelvir plus ritonavir within 5 days of symptom onset in adults with COVID-19 who do not require oxygen and are immunocompromised; or are at particularly high risk of severe disease on the basis of advanced age and multiple risk factors.**<br><br>Decisions about the appropriateness of nirmatrelvir plus ritonavir should be based on the individual’s risk of severe disease, including their age, presence of multiple risk factors, and vaccination status (including number of doses and time since last dose/ or timing of most recent infection).<br><br>Available research does not currently provide enough evidence to determine the benefits of nirmatrelvir plus ritonavir in specific subgroups of patients. In the absence of definitive evidence, the Taskforce has arrived at a consensus recommendation based on their combined clinical expertise to guide clinical decisions about which patients are most likely to benefit from nirmatrelvir plus ritonavir.

	There is no evidence evaluating the effectiveness of nirmatrelvir plus ritonavir in individuals who have received any COVID-19 vaccine. Given this, and the lower risk of deterioration in these people, it is less likely that nirmatrelvir plus ritonavir will be of benefit in individuals who have received a vaccine dose or had a SARS-CoV-2 infection in the past 6 months, unless the patient is immunocompromised.<br><br>There is limited evidence on the effectiveness of nirmatrelvir plus ritonavir in immunosuppressed patients. However, given the likely higher risk of deterioration in these patients, and the absence of reasons to believe otherwise, it is likely that nirmatrelvir plus ritonavir will be beneficial for immunocompromised patients.<br><br>Immunocompromising conditions include:<br><br>• Primary or acquired immunodeficiency<br>o Haematologic neoplasms: leukaemias, lymphomas, myelodysplastic syndromes<br>o Post-transplant: solid organ (on immunosuppressive therapy), haematopoietic stem cell transplant (within 24 months)<br>o Immunocompromised due to primary or acquired (HIV/AIDS) immunodeficiency<br>o Other significantly immunocompromising conditions<br>• Immunosuppressive therapy (current or recent)<br>o Chemotherapy, whole body radiotherapy or total lymphoid irradiation<br>o High-dose corticosteroids (≥ 20 mg of prednisone per day, or equivalent) for ≥ 14 days<br>o All biological disease-modifying anti-rheumatic drugs (DMARDs) and most other (e.g. conventional synthetic) DMARDs[20]<br><br>In the absence of reliable evidence, the Taskforce has developed this recommendation based on their clinical expertise and experience.<br><br>Nirmatrelvir plus ritonavir is currently listed within the Australian Pharmaceutical Benefits Scheme.[25]
**Regdanvimab (Regkirona™)**	
**Conditional recommendation**	**Consider using regdanvimab within 7 days of symptom onset in unvaccinated* adults with COVID-19 who do not require oxygen and who have one or more risk factors for disease progression.**<br><br>**Where infection with Omicron BA.1, BA.2, BA.4 or BA.5 is confirmed or considered likely, use of regdanvimab should only be considered where other treatments are not suitable or available.**<br><br>**Within the patient population for which regdanvimab is recommended for use (see Remark), decisions about the appropriateness of treatment with regdanvimab should be based on the individual's risk of severe disease, on the basis of age and multiple risk factors, COVID-19 vaccination status and time since vaccination.**

	**** Individuals who had received one or more doses of SARS-CoV-2 vaccine were excluded from the trial. The efficacy of regdanvimab is unclear in partially or fully vaccinated individuals. Additional recommendations for other patient groups are currently under development and will be included in a future version of the guideline.***
<u>Remark</u>	*While the clinical evidence supports use of regdanvimab to treat mild COVID-19, there is no clinical evidence to evaluate its effectiveness against the Omicron variant or BA.1, BA.2, BA.4 or BA.5 sub-variants. The Taskforce is aware of in vitro data that suggest potentially reduced efficacy against these variants and while the clinical implications of this are not certain, given the availability of other treatments, where infection with Omicron BA.2, BA.4 or BA.5 is confirmed or considered likely, use of regdanvimab should not be considered unless other treatments are unsuitable or unavailable.*<br><br>In patients with confirmed COVID-19 who do not require oxygen, regdanvimab (Regkirona) probably decreases the risk of hospitalisation and supplemental oxygen, and improves clinical recovery if taken within 7 days of onset of symptoms.<br><br>Results are based on a two sequential trials (Part 1 and Part 2) comparing regdanvimab with placebo in 1629 unvaccinated adults with PCR-confirmed COVID-19 and mild illness[26,27]. Within these trials, participants were treated with a single 40 mg/kg dose of regdanvimab, administered as an intravenous infusion over 60 minutes.<br><br>Although the trial did not limit participation to individuals who were at high risk of progression, the benefit of regdanvimab is likely to be greatest in those with the greatest risk of severe disease. Of the 1315 participants included in Part 2 of the trial, two thirds had one or more risk factors for disease progression, defined as individuals who met at least one of the following criteria:<br>• Age > 50 years<br>• BMI > 30 kg/m2<br>• Cardiovascular disease, including hypertension<br>• Chronic lung disease, including asthma<br>• Type 1 or type 2 diabetes<br>• Chronic kidney disease, including those on dialysis<br>• Chronic liver disease<br>• Immunocompromised (e.g. cancer treatment, bone marrow or organ transplantation, immune deficiencies, HIV, sickle cell anaemia, thalassaemia and prolonged use of immune weakening medications)<br><br>Pregnant and breastfeeding women and children and adolescents were not included in the trial.<br><br>The efficacy of regdanvimab in vaccinated or immunocompromised patients is unknown.

	The Taskforce is aware of concerns about the potential for decreased effectiveness of regdanvimab against the BA.2, BA.4 and BA.5 Omicron sub-variants, based on in vitro data. We will continue to monitor for further evidence as it emerges and update this recommendation when definitive evidence becomes available.<br><br>As of 31 July 2022, the Taskforce has made conditional recommendations supporting the use of sotrovimab, casirivimab plus imdevimab, tixagevimab plus cilgavimab, nirmatrelvir plus ritonavir, remdesivir, regdanvimab and molnupiravir in adult outpatients with mild COVID-19. The relative effectiveness of these agents, or the effectiveness of these agents when used in combination, is unclear.<br><br>*This is a high priority recommendation and will be updated as soon as new evidence becomes available.*
**Consensus recommendation**<br><br>***Remark***	**In addition to at-risk unvaccinated adults, also consider using regdanvimab within 7 days of symptom onset in adults with COVID-19 who do not require oxygen and are immunocompromised; or are at particularly high risk of severe disease on the basis of advanced age and multiple risk factors.**<br><br>**Where infection with Omicron BA.1, BA.2, BA.4 or BA.5 is confirmed or considered likely, use of regdanvimab should only be considered where other treatments are not suitable or available.**<br><br>*While the clinical evidence supports use of regdanvimab to treat mild COVID-19, there is no clinical evidence to evaluate its effectiveness against the Omicron variant or BA.1, BA.2, BA.4 or BA.5 sub-variants. The Taskforce is aware of in vitro data that suggest potentially reduced efficacy against these variants and while the clinical implications of this are not certain, given the availability of other treatments, where infection with Omicron BA.2, BA.4 or BA.5 is confirmed or considered likely, use of regdanvimab should not be considered unless other treatments are unsuitable or unavailable.*<br><br>Available research does not currently provide enough evidence to determine the benefits of regdanvimab (Regkirona) in specific subgroups of patients. In the absence of definitive evidence, the Taskforce has arrived at a consensus recommendation based on their combined clinical expertise to guide clinical decisions about which patients are most likely to benefit from regdanvimab.<br><br>Decisions about the appropriateness of regdanvimab should be based on the individual's risk of severe disease, including their age, presence of multiple risk factors, and vaccination status (including number of doses and time since last dose/ or timing of most recent infection).<br><br>There is no evidence evaluating the effectiveness of regdanvimab in individuals who have received any COVID-19 vaccine. Given this, and the lower risk of deterioration in these patients, it is unlikely that regdanvimab will be particularly valuable in individuals who have received a vaccine dose or had a SARS-CoV-2 infection in the past 6 months, unless the patient is immunocompromised.

	There is limited evidence on the effectiveness of regdanvimab in immunocompromised patients. However, given the likely higher risk of deterioration in these patients, and the absence of reasons to believe otherwise, it is likely that regdanvimab will be beneficial for immunocompromised patients.<br><br>Immunocompromising conditions include:<br><br>• Primary or acquired immunodeficiency<br>○ Haematologic neoplasms: leukaemias, lymphomas, myelodysplastic syndromes<br>○ Post-transplant: solid organ (on immunosuppressive therapy), haematopoietic stem cell transplant (within 24 months)<br>○ Immunocompromised due to primary or acquired (HIV/AIDS) immunodeficiency<br>○ Other significantly immunocompromising conditions<br>• Immunosuppressive therapy (current or recent)<br>○ Chemotherapy, whole body radiotherapy or total lymphoid irradiation<br>○ High-dose corticosteroids (≥ 20 mg of prednisone per day, or equivalent) for ≥ 14 days<br>○ All biological disease-modifying anti-rheumatic drugs (DMARDs) and most other (e.g. conventional synthetic) DMARDs[20]<br><br>In the absence of reliable evidence, the Taskforce has developed this recommendation based on their clinical expertise and experience.<br><br>The Taskforce is aware of concerns about the potential for decreased effectiveness of regdanvimab against the BA.4 and BA.5 Omicron sub-variants, based on in vitro data. We will update this recommendation when definitive evidence becomes available.<br><br>*This is a high priority recommendation and will be updated as soon as new evidence becomes available.*
**Remdesivir (Veklury™)**	
**Conditional recommendation**	**Consider using remdesivir within 7 days of symptom onset in unvaccinated* adults with COVID-19 who do not require oxygen and who have one or more risk factors for disease progression.**<br><br>**Within the patient population for which remdesivir is conditionally recommended for use (see Remark), decisions about the appropriateness of remdesivir should be based on the individual’s risk of severe disease, including their age, presence of multiple risk factors, and vaccination status (including number of doses and time since last dose/ or timing of most recent infection).**<br><br>**** Individuals who had received one or more doses of SARS-CoV-2 vaccine were excluded from the trial. The efficacy of remdesivir is unclear in individuals who are up-to-date with vaccination or partially vaccinated. See consensus recommendation for guidance on use of remdesivir in vaccinated adults or in immunocompromised patients regardless of vaccination status.***
Remark	In adults with confirmed COVID-19 who do not require oxygen, remdesivir probably decreases the risk of hospitalisation if taken within 7 days of onset of symptoms.

	Results are based on a single trial[28], in which unvaccinated adults were administered three intravenous doses of remdesivir on consecutive days (200 mg on day 1, followed by 100 mg on days 2 and 3). Based on the inclusion criteria for this trial, risk factors for disease progression include the following:<br><br>• Age ≥ 60 years<br>• Diabetes<br>• Obesity (BMI ≥ 30 kg/m2)<br>• Chronic kidney disease (any stage)<br>• Cardiovascular or cerebrovascular disease (coronary artery disease, congenital heart disease, heart failure, cardiomyopathy or history of stroke)<br>• Hypertension (systemic or pulmonary)<br>• Chronic liver disease<br>• Chronic lung disease (chronic obstructive pulmonary disease, moderate-severe asthma, cystic or pulmonary fibrosis)<br>• Sickle cell disease<br>• Current cancer<br>• Immunocompromised state (no definition provided)<br><br>Pregnant and breastfeeding women were not included in the trial. Eight paediatric patients aged 12–18 years were included, none of whom progressed to hospitalisation or death.<br><br>The efficacy of remdesivir in vaccinated and immunocompromised patients is unknown.<br><br>As of 07 March 2022, the Taskforce has made conditional recommendations supporting the use of sotrovimab, casirivimab plus imdevimab, nirmatrelvir plus ritonavir, remdesivir and molnupiravir in adult outpatients with mild COVID-19. The relative effectiveness of these agents, or the effectiveness of these agents when used in combination, is unclear.<br><br>*This is a high priority recommendation and will be updated as soon as new evidence becomes available.*
**Consensus recommendation**<br><br>***Remark***	**In addition to at-risk unvaccinated adults, also consider using remdesivir within 7 days of symptom onset in adults with COVID-19 who do not require oxygen and are immunocompromised; or are at particularly high risk of severe disease on the basis of advanced age and multiple risk factors.**<br><br>Decisions about the appropriateness of remdesivir should be based on the individual's risk of severe disease, including their age, presence of multiple risk factors, and vaccination status (including number of doses and time since last dose/ or timing of most recent infection).

	Available research does not currently provide enough evidence to determine the benefits of remdesivir in specific subgroups of patients. In the absence of definitive evidence, the Taskforce has arrived at a consensus recommendation based on their combined clinical expertise to guide clinical decisions about which adults are most likely to benefit from remdesivir.<br><br>There is no evidence evaluating the effectiveness of remdesivir in individuals who have received any COVID-19 vaccine, a low likelihood of development of severe disease, and a small risk of adverse events. Given this, and the lower risk of deterioration in these individuals, it is less likely that remdesivir will have a significant treatment benefit in individuals who have received a vaccine dose or had a SARS-CoV-2 infection in the past 6 months, unless the individual is immunocompromised.<br><br>There is limited evidence on the effectiveness of remdesivir in immunosuppressed patients. However, given the likely higher risk of deterioration in these patients, and the absence of reasons to believe otherwise, it is likely that remdesivir will be beneficial for immunosuppressed patients.<br><br>Immunocompromising conditions include:<br><br>• Primary or acquired immunodeficiency<br>o Haematologic neoplasms: leukaemias, lymphomas, myelodysplastic syndromes<br>o Post-transplant: solid organ (on immunosuppressive therapy), haematopoietic stem cell transplant (within 24 months)<br>o Immunocompromised due to primary or acquired (HIV/AIDS) immunodeficiency<br>o Other significantly immunocompromising conditions<br>• Immunosuppressive therapy (current or recent)<br>o Chemotherapy, whole body radiotherapy or total lymphoid irradiation<br>o High-dose corticosteroids (≥ 20 mg of prednisone per day, or equivalent) for ≥ 14 days<br>o All biological disease-modifying anti-rheumatic drugs (DMARDs) and most other (e.g. conventional synthetic) DMARDs [5]<br><br>In the absence of reliable evidence, the Taskforce has developed this recommendation based on their clinical expertise and experience.
**Sotrovimab (Xevudy™)**	
**Conditional recommendation**	**Consider using sotrovimab within 5 days of symptom onset in unvaccinated* adults with COVID-19 who do not require oxygen and who have one or more risk factors for disease progression.**<br><br>**Where infection with Omicron BA.2, BA.4 or BA.5 is confirmed or considered likely, use of sotrovimab should only be considered where other treatments are not suitable or available.**

	**Within the patient population for which sotrovimab is conditionally recommended for use (see Remark), decisions about the appropriateness of sotrovimab should be based on the individual's risk of severe disease, including their age, presence of multiple risk factors, and vaccination status (including number of doses and time since last dose/ or timing of most recent infection).**<br><br>**** Individuals who had received one or more doses of SARS-CoV-2 vaccine were excluded from the trial. The efficacy of sotrovimab is unclear in individuals who are up-to-date with vaccination or partially vaccinated. See consensus recommendation for guidance on use of sotrovimab in vaccinated adults or in immunocompromised patients regardless of vaccination status.***
<u>Remark</u>	*While the clinical evidence supports use of sotrovimab to treat mild COVID-19, there is no clinical evidence to evaluate its effectiveness against the Omicron variant or BA.1, BA.2, BA.4 or BA.5 sub-variants. The Taskforce is aware of in vitro data that suggest potentially reduced efficacy against these variants and while the clinical implications of this are not certain, given the availability of other treatments, where infection with Omicron BA.2, BA.4 or BA.5 is confirmed or considered likely, use of sotrovimab should not be considered unless other treatments are unsuitable or unavailable.*<br><br>In patients with confirmed COVID-19 who do not require oxygen, sotrovimab probably decreases the risk of hospitalisation if taken within 5 days of onset of symptoms.<br><br>Results are based on the COMET-ICE trial[29], in which unvaccinated adults were treated with a single one-hour intravenous infusion of 500 mg of sotrovimab. Based on the inclusion criteria for this trial, risk factors for disease progression include the following:<br><br>• Diabetes (requiring medication)<br>• Obesity (BMI ≥ 30 kg/m2)<br>• Chronic kidney disease (i.e. eGFR < 60 by MDRD)<br>• Congestive heart failure (NYHA class II or greater)<br>• Chronic obstructive pulmonary disease (history of chronic bronchitis, chronic obstructive lung disease, or emphysema with dyspnoea on physical exertion)<br>• Moderate-to-severe asthma (requiring an inhaled steroid to control symptoms or prescribed a course of oral steroids in the previous 12 months)<br>• Age ≥ 55 years<br><br>Pregnant and breastfeeding women and children and adolescents were not included in the trial. However trials are underway in which children over 12 years of age are eligible for inclusion (OPTIMISE-C19, NCT04913675).<br><br>The efficacy of sotrovimab in vaccinated or immunocompromised patients is unknown.

	As of 28 February 2022, the Taskforce has made conditional recommendations supporting the use of sotrovimab, casirivimab plus imdevimab, nirmatrelvir plus ritonavir, remdesivir and molnupiravir in adult outpatients with mild COVID-19. The relative effectiveness of these agents, or the effectiveness of these agents when used in combination, is unclear.<br><br>*This is a <u>high priority</u> recommendation and will be updated as soon as new evidence becomes available.*
**Consensus recommendation**<br><br><br><br><br>***<u>Remark</u>***	**In addition to at-risk unvaccinated adults, also consider using sotrovimab within 5 days of symptom onset in adults with COVID-19 who do not require oxygen and:**<br>• **are immunocompromised regardless of vaccination status; or**<br>• **who are not up-to-date with vaccination and who are at high risk of disease on the basis of age and multiple risk factors**<br><br>Where infection with Omicron BA.2, BA.4 or BA.5 is confirmed or considered likely, use of sotrovimab should only be considered where other treatments are not suitable or available.<br><br>*While the clinical evidence supports use of sotrovimab to treat mild COVID-19, there is no clinical evidence to evaluate its effectiveness against the Omicron variant or BA.1, BA.2, BA.4 or BA.5 sub-variants. The Taskforce is aware of in vitro data that suggest potentially reduced efficacy against these variants and while the clinical implications of this are not certain, given the availability of other treatments, where infection with Omicron BA.2, BA.4 or BA.5 is confirmed or considered likely, use of sotrovimab should not be considered unless other treatments are unsuitable or unavailable.*<br><br>Decisions about the appropriateness of sotrovimab should be based on the individual's risk of severe disease, including their age, presence of multiple risk factors, and vaccination status (including number of doses and time since last dose/ or timing of most recent infection).<br><br>Available research does not currently provide enough evidence to determine the benefits of sotrovimab in specific subgroups of patients. In the absence of definitive evidence, the Taskforce has arrived at a consensus recommendation based on their combined clinical expertise to guide clinical decisions about which adults are most likely to benefit from sotrovimab.<br><br>There is no evidence evaluating the effectiveness of sotrovimab in individuals who have received any COVID-19 vaccine, a low likelihood of development of severe disease, and a small risk of adverse events. Given this, and the lower risk of deterioration in these individuals, it is less likely that sotrovimab will have a significant treatment benefit in people who have received a vaccine dose or had a SARS-CoV-2 infection in the past 6 months, unless the individual is immunocompromised.<br><br>There is no evidence on the effectiveness of sotrovimab in immunosuppressed patients. However, given the likely higher risk of deterioration in these individuals, and the absence of reasons to believe otherwise, it is likely that sotrovimab will be beneficial for immunocompromised individuals.<br>Immunocompromising conditions include:

	• Primary or acquired immunodeficiency<br>○ Haematologic neoplasms: leukaemias, lymphomas, myelodysplastic syndromes<br>○ Post-transplant: solid organ (on immunosuppressive therapy), haematopoietic stem cell transplant (within 24 months)<br>○ Immunocompromised due to primary or acquired (HIV/AIDS) immunodeficiency<br>○ Other significantly immunocompromising conditions<br>• Immunosuppressive therapy (current or recent)<br>○ Chemotherapy, whole body radiotherapy or total lymphoid irradiation<br>○ High-dose corticosteroids (≥ 20 mg of prednisone per day, or equivalent) for ≥ 14 days<br>○ All biological disease-modifying anti-rheumatic drugs (DMARDs) and most other (e.g. conventional synthetic) DMARDs[20]<br><br>In the absence of reliable evidence, the Taskforce has developed this recommendation based on their clinical expertise and experience.
**Tixagevimab plus cilgavimab (Evusheld™)**	
**Conditional recommendation**	**Consider using tixagevimab plus cilgavimab within 5 days of symptom onset in unvaccinated* adults with COVID-19 who do not require oxygen and who have one or more risk factors for disease progression.**<br><br>**Within the patient population for which tixagevimab plus cilgavimab is conditionally recommended for use (see Remark), decisions about the appropriateness of treatment with tixagevimab plus cilgavimab should be based on the individual's risk of severe disease, including their age, presence of multiple risk factors and vaccination status (including number of doses and time since last dose/ or timing of most recent infection).**<br><br>**** Individuals who had received one or more doses of SARS-CoV-2 vaccine were excluded from the trial. The efficacy of tixagevimab plus cilgavimab is unclear in individuals who are up-to-date with vaccination or partially vaccinated. See consensus recommendation for guidance on use of tixagevimab plus cilgavimab in vaccinated patients or in immunocompromised patients regardless of vaccination status.***
Remark	In adults with confirmed COVID-19 who do not require oxygen, tixagevimab plus cilgavimab probably decreases the risk of hospitalisation if taken within 5 days of onset of symptoms.<br>Results are based on the TACKLE trial[30], in which 903 unvaccinated adults were treated with a single dose of 600 mg Evusheld consisting of two intramuscular injections (300 mg tixagevimab and 300 mg cilgavimab). Based on the inclusion criteria for this trial, risk factors for disease progression include the following:<br>• Age ≥ 65 years<br>• Diabetes<br>• Obesity (BMI ≥ 30 kg/m2)<br>• Chronic kidney disease<br>• Cardiovascular disease (including history of stroke)<br>• Hypertension

	• Chronic liver disease<br>• Chronic lung disease or moderate-to-severe asthma<br>• Sickle cell disease<br>• Smoking (current and former)<br>• Cancer<br>• Immunocompromised state (from solid organ transplant, blood or bone marrow transplant, immune deficiencies, HIV, use of corticosteroids, or use of other immunosuppressive medicines)<br><br>Pregnant & breastfeeding women and children & adolescents were not included in the trial.<br><br>The efficacy of tixagevimab plus cilgavimab in vaccinated and immunocompromised patients is unknown.<br><br>As of 24 March 2022, the Taskforce has made conditional recommendations supporting the use of sotrovimab, casirivimab plus imdevimab, tixagevimab plus cilgavimab, nirmatrelvir plus ritonavir, remdesivir and molnupiravir in adult outpatients with mild COVID-19. The relative effectiveness of these agents, or the effectiveness of these agents when used in combination, is unclear.<br><br>As of 24 March 2022, the Therapeutic Goods Administration has not approved tixagevimab plus cilgavimab for this indication.<br><br>The concurrent use of two or more monoclonal antibodies should be avoided except where co-formulated.<br><br>The Taskforce is aware of concerns about the potential for decreased effectiveness of tixagevimab plus cilgavimab against the BA.4 and BA.5 Omicron sub-variants, based on in vitro data, and about the suggestion of increased rates of cardiac events in intervention arms of the TACKLE and STORMCHASER trials[30,31]. We will continue to monitor for further evidence as it emerges and update this recommendation when definitive evidence becomes available.<br><br>*This is a high priority recommendation and will be updated as soon as new evidence becomes available.*
**Consensus recommendation**<br><br><br><br>***Remark***	**In addition to at-risk unvaccinated adults, also consider using tixagevimab plus cilgavimab within 5 days of symptom onset in adults with COVID-19 who do not require oxygen and are immunocompromised; or are at particularly high risk of severe disease on the basis of advanced age and multiple risk factors.**<br><br>Available research does not currently provide enough evidence to determine the benefits of tixagevimab plus cilgavimab in specific subgroups of patients. In the absence of definitive evidence, the Taskforce has arrived at a consensus recommendation based on their combined clinical expertise to guide clinical decisions about which patients are most likely to benefit from tixagevimab plus cilgavimab. Decisions about the appropriateness of tixagevimab plus cilgavimab should be based on the individual's risk of severe disease, including their age, presence of multiple risk factors, and vaccination status (including number of doses and time since last dose/ or timing of most recent infection).

<table>
<tr>
<td></td>
<td>
There is no evidence evaluating the effectiveness of tixagevimab plus cilgavimab in individuals who have received any COVID-19 vaccine. Given this, and the lower risk of deterioration in these people, it is unlikely that tixagevimab plus cilgavimab will be particularly valuable in individuals who have received a vaccine dose or had a SARS-CoV-2 infection in the past 6 months, unless the patient is immunocompromised.
There is limited evidence on the effectiveness of tixagevimab plus cilgavimab in immunosuppressed patients. However, given the likely higher risk of deterioration in these patients, and the absence of reasons to believe otherwise, it is likely that tixagevimab plus cilgavimab will be beneficial for immunosuppressed patients.
Immunocompromising conditions include:

Primary or acquired immunodeficiency

Haematologic neoplasms: leukaemias, lymphomas, myelodysplastic syndromes
Post-transplant: solid organ (on immunosuppressive therapy), haematopoietic stem cell transplant (within 24 months)
Immunocompromised due to primary or acquired (HIV/AIDS) immunodeficiency
Other significantly immunocompromising conditions

Immunosuppressive therapy (current or recent)

Chemotherapy, whole body radiotherapy or total lymphoid irradiation
High-dose corticosteroids (≥ 20 mg of prednisone per day, or equivalent) for ≥ 14 days
All biological disease-modifying anti-rheumatic drugs (DMARDs) and most other (e.g. conventional synthetic) DMARDs[20]

In the absence of reliable evidence, the Taskforce has developed this recommendation based on their clinical expertise and experience.
The Taskforce is aware of concerns about the potential for decreased effectiveness of tixagevimab plus cilgavimab against the BA.4 and BA.5 Omicron sub-variants, based on in vitro data. We will update this recommendation when definitive evidence becomes available
This is a <u>high priority</u> recommendation and will be updated as soon as new evidence becomes available.
</td>
</tr>
</table>

BMI – body mass index; CKD – chronic kidney disease; CPAP – continuous positive airway pressure; CYP3A – cytochrome P450 3A4; eGFR – estimated glomerular filtration rate; EPIC-SR – Evaluation of Protease Inhibition for COVID-19 in Standard-Risk Patients Trial; EUA – emergency use authorisation; FDA – U.S Food and Drug Administration; HIV/AIDS – human immunodeficiency virus / acquired immune deficiency syndrome; MDRD – Modification of Diet in Renal Disease study equation; NCT04913675 – Intramuscular VIR-7831 (Sotrovimab) for Mild/Moderate COVID-19 substudy; NYHA – New York Heart Association; OPTIMISE-C19 – The UPMC Optimizing Treatment and Impact of Monoclonal antIbodieS Through Evaluation for COVID-19 Trial; PCR – polymerase chain reaction; PI – product information; SARS-CoV-2 – severe acute respiratory syndrome coronavirus 2; SSRN – Social Science Research Network; TGA – Therapeutic Goods Administration

Table 4. Recommendations for pre- and post-exposure prophylaxis (guideline section 7)

**Hydroxychloroquine (pre-exposure prophylaxis)**	
**Not recommended**	**For healthcare workers with no active COVID-19, do not use hydroxychloroquine for pre-exposure prophylaxis outside of randomised trials with appropriate ethical approval.**
<u>Remark</u>	Trials are needed in special populations, including children and adolescents, pregnant and breastfeeding women, older people living with frailty and those receiving palliative care. Until further evidence is available, do not use hydroxychloroquine for pre-exposure prophylaxis in these populations unless they are eligible to be enrolled in trials.<br><br>*This is a <u>low priority</u> recommendation and we do not expect to update it in the immediate future, however we continue to conduct daily searches for new evidence.*
**Tixagevimab plus cilgavimab (pre-exposure prophylaxis)**	
**Consensus recommendation**	**Do not routinely use tixagevimab plus cilgavimab as pre-exposure prophylaxis, however use may be considered in exceptional circumstances, in individuals who are severely immunocompromised.**<br><br>**Given the limited evidence of benefit or safety, small effect sizes and absence of evidence evaluating the effectiveness of tixagevimab plus cilgavimab for prevention of infection by SARS-CoV-2 variants of concern, rigorous data collection should be undertaken on indications and key outcomes for adults who receive pre-exposure prophylaxis with tixagevimab plus cilgavimab.**
<u>Remark</u>	Evidence regarding the potential effectiveness of tixagevimab plus cilgavimab in preventing SARS-CoV-2 infection is very limited, with small sample sizes and low event rates. Given the limited evidence, the panel was not able to reach consensus on whether use of tixagevimab plus cilgavimab should be recommended for pre-exposure prophylaxis. However some members of the panel felt that use could be considered in exceptional circumstances for people who are at high risk of progression, specifically those who are severely immunocompromised.<br><br>Results are based on the PROVENT trial[32], in which 5197 unvaccinated adults were administered a single 300 mg dose of Evusheld consisting of two intramuscular injections (150 mg tixagevimab and 150 mg cilgavimab). Included participants required an increased risk for inadequate response to vaccination, defined within the trial as:<br><br>• ≥ 60 years old<br>• BMI ≥ 30 kg/m2<br>• Congestive heart failure<br>• Chronic obstructive pulmonary disease<br>• Chronic kidney disease (eGFR < 30 mL/min)<br>• Chronic liver disease

	• Immunocompromised state from solid organ transplant, blood or bone marrow transplant, immune deficiencies, HIV, use of corticosteroids or other immunosuppressive medicines<br>• Intolerant of vaccine<br><br>OR be at increased risk for SARS-CoV-2 infection based on location or circumstance, defined within the trial as:<br><br>• Healthcare workers<br>• Workers in industrial settings shown to have been at high-risk for SARS-CoV-2 transmission (e.g. meatpacking plants)<br>• Military personnel residing or working in high-density settings<br>• Students living in dormitory settings<br>• Others living in similar settings of similar close or high-density proximity<br><br>Pregnant and breastfeeding women and children and adolescents were not included in the trial.<br><br>A total of 18.4% of participants had received COVID-19 vaccination between time of tixagevimab plus cilgavimab administration and data cut-off (12.2% tixagevimab plus cilgavimab, 30.7% placebo). Results were not reported separately for this subgroup.<br>*In vitro* data varies around whether tixagevimab plus cilgavimab maintains efficacy against the Omicron variant; however the study was conducted before the Omicron variant was prevalent and there are no clinical data regarding the effectiveness of tixagevimab plus cilgavimab specific to the Omicron variant.<br><br>The Taskforce is aware of concerns about the suggestion of increased rates of cardiac events in intervention arms of the TACKLE and STORMCHASER trials, and will continue to monitor for further evidence as it emerges.<br><br>In the absence of reliable evidence, the Taskforce has developed this recommendation based on their clinical expertise and experience.<br><br>*This is a high priority recommendation and will be updated as soon as new evidence becomes available.*
**Casirivimab plus imdevimab (Ronapreve™; post-exposure prophylaxis)**	
**Conditional recommendation**<br><br><br><br>***Remark***	**Consider using subcutaneous casirivimab plus imdevimab as prophylaxis in seronegative or polymerase chain reaction-negative close household contacts of individuals with confirmed COVID-19.**<br><br>**Where Omicron is likely to be the dominant circulating variant, use of casirivimab plus imdevimab as post-exposure prophylaxis is unlikely to be effective and should only be used in exceptional circumstances.**<br><br>*While the clinical evidence supports use of casirivimab plus imdevimab as post-exposure prophylaxis, there is no clinical evidence to evaluate its effectiveness against the Omicron variant or BA.1, BA.2, BA.4 and BA.5 sub-variants. The Taskforce is aware of* in vitro *data*

	that suggest potentially reduced efficacy against these variants and while the clinical implications of this are not certain, the use of casirivimab plus imdevimab as post-exposure prophylaxis is unlikely to be effective and should only be used in exceptional circumstances.<br><br>The use of prophylactic casirivimab plus imdevimab probably reduces the risk of symptomatic and asymptomatic COVID-19 infection in seronegative household contacts of individuals with confirmed COVID-19 when used within 4 days of exposure. In settings for which serology testing is not readily available, consider using in unvaccinated adult household contacts who have risk factors for developing severe disease, return a negative polymerase chain reaction (PCR) result and are considered unlikely to have had previous SARS-CoV-2 infection.<br><br>Results are based on one trial, in which 1200 mg of casirivimab plus imdevimab (600 mg of each) was administered subcutaneously to close household contacts of individuals with confirmed COVID-19.[33] Participants were healthy individuals aged 12 years or older who were seronegative for SARS-CoV-2 antibodies at the time of treatment.<br><br>The following should be considered when determining the appropriateness of treatment:<br><br>• Vaccinated individuals were excluded from the trial—the ability of casirivimab plus imdevimab to prevent COVID-19 infection in this population is not known.<br>• The effectiveness of casirivimab plus imdevimab in preventing COVID-19 infection in patients who are seropositive to SARS-CoV-2 antibodies or who are immunosuppressed is not known.<br>• In individuals who go on to develop COVID-19, the impact of prophylactic casirivimab plus imdevimab on subsequent outcomes of interest, such as hospitalisation, requirement of supplemental oxygen or death, is not known.<br><br>The Taskforce recognises that subcutaneous casirivimab plus imdevimab may be administered to household contacts who were PCR-negative at the time of testing, but become PCR-positive by the time of receiving casirivimab plus imdevimab. Although the Taskforce does not currently recommend casirivimab plus imdevimab for PCR-positive individuals with asymptomatic or mildly symptomatic COVID-19, this treatment is unlikely to result in harm.<br><br>This trial was conducted in a population exposed to a mixture of SARS-CoV-2 variants, but before the emergence and dominance of the Delta variant. The effectiveness of casirivimab plus imdevimab in populations exposed to the Delta variant of SARS-CoV-2 has not been established.<br><br>The Taskforce is aware of concerns about the potential for decreased effectiveness of casirivimab plus imdevimab against the Omicron variant, based on *in vitro* data. We will update this recommendation as definitive evidence becomes available.<br><br>*This is a <u>high priority</u> recommendation and will be updated as soon as new evidence becomes available.*

Hydroxychloroquine (post-exposure prophylaxis)	
**Not recommended**	**For people exposed to individuals with SARS-CoV-2 infection, do not use hydroxychloroquine for post-exposure prophylaxis outside of randomised trials with appropriate ethical approval.**
Remark	Trials are needed in special populations, including children and adolescents, pregnant and breastfeeding women, older people living with frailty and those receiving palliative care. Until further evidence is available, do not use hydroxychloroquine for post-exposure prophylaxis in these populations unless they are eligible to be enrolled in trials.<br><br>*This is a low priority recommendation and we do not expect to update it in the immediate future, however we continue to conduct daily searches for new evidence.*
**Tixagevimab plus cilgavimab (post-exposure prophylaxis)**	
**Only in research settings**	**For people exposed to individuals with SARS-CoV-2 infection, do not use tixagevimab plus cilgavimab for post-exposure prophylaxis outside of randomised trials with appropriate ethical approval.**
Remark	Evidence suggests that administration of tixagevimab plus cilgavimab has little impact on preventing SARS-CoV-2 infection when administered as post-exposure prophylaxis in people who have been in close contact with an individual with confirmed COVID-19.<br><br>*This is a moderate priority recommendation and will be updated when new evidence becomes available that is likely to impact the direction or strength of the recommendation.*

SARS-CoV-2 – severe acute respiratory syndrome coronavirus 2

Table 5. Recommendations for respiratory support in non-pregnant adults with COVID-19 (guideline section 8)

**Guiding principles of care**	
**Consensus recommendation**	**For patients with COVID-19 receiving respiratory support, use single and negative pressure rooms wherever possible. If none are available, other alternatives are single rooms, or shared ward spaces with cohorting of confirmed COVID-19 patients. Ensure contact, droplet and airborne precautions are in place. Healthcare workers should be fully vaccinated and wearing fit-tested N95 masks.**
Remark	The additional relative risk of infection to healthcare workers associated with specific oxygen therapies and respiratory support is uncertain but is thought to add minimal additional risk in an environment where transmission of infection with COVID-19 is already high.
**Continuous positive airway pressure (CPAP)**	
**Conditional recommendation**	**For patients with COVID-19 who have hypoxaemic respiratory failure and are unable to maintain oxygen saturations within target range despite oxygen delivery by nasal prongs or mask, consider using continuous positive airway pressure (CPAP).**<br><br>**The evidence suggests that CPAP therapy is preferred for patients with persistent hypoxaemia associated with COVID-19 (defined as requiring an $FiO_2 \geq 0.4$ to maintain oxygen saturation in their target range). Adjust continuous positive airway pressure as required, most patients require pressures of 10 to 12 cmH2O. Excessive pressures may increase the risk of pneumothorax. Titrate oxygen to maintain oxygen saturation in the target range. There is currently insufficient direct evidence available to support the use of bilevel positive pressure support in the setting of COVID-19.**<br><br>**If CPAP is not available or not tolerated, consider high-flow nasal oxygen (HFNO) as an alternative using the same safety parameters.**<br><br>**Patients receiving CPAP (and/or HFNO) for COVID-19, monitor closely at all times and liaise with ICU in case of deterioration. Do not delay endotracheal intubation and invasive mechanical ventilation in patients with COVID-19 who are deteriorating despite optimised, less invasive respiratory therapies.**
**Respiratory management of the deteriorating patient**	
**Consensus recommendation**	**Do not delay endotracheal intubation and mechanical ventilation in patients with COVID-19 who are deteriorating despite optimised, less invasive respiratory therapies.**
Remark	Patients can deteriorate rapidly 5 to 10 days after onset of symptoms.<br><br>The net clinical benefit for each patient should be considered on a case-by-case basis, as factors such as frailty, advanced illness or comorbidity may lessen the benefit and increase potential harms.<br><br>Decisions around proceeding to more invasive forms of therapy should be discussed with the patient or their substitute / medical treatment decision-maker. The goals of patient care need to balance the preferences and values of the patient, based on discussion and

	an advance care directive or plan if available, and consideration of the patient's expected short- and long-term responses to more invasive forms of treatment.<br><br>*This is a <u>low priority</u> recommendation and we do not expect to update it in the immediate future, however we continue to conduct daily searches for new evidence.*
**Videolaryngoscopy**	
**Conditional recommendation**	**In adults with COVID-19 undergoing endotracheal intubation, consider using videolaryngoscopy over direct laryngoscopy if available and the operator is trained in its use.**
<u>Remark</u>	*This is a <u>low priority</u> recommendation and we do not expect to update it in the immediate future, however we continue to conduct daily searches for new evidence.*
**Neuromuscular blockers**	
**Conditional recommendation against**	**For mechanically ventilated adults with COVID-19 and moderate-to-severe acute respiratory distress syndrome, do not routinely use continuous infusions of neuromuscular blocking agents (NMBAs).**
<u>Remark</u>	However, if protective lung ventilation cannot be achieved, consider using NMBAs for up to 48 hours. If indicated, consider cisatracurium as first-line agent, if cisatracurium is not available alternatives include atracurium or vecuronium by infusion.<br><br>*This is a <u>low priority</u> recommendation and we do not expect to update it in the immediate future, however we continue to conduct daily searches for new evidence.*
**Positive end-expiratory pressure (PEEP)**	
**Consensus recommendation**	**For mechanically ventilated adults with COVID-19 and moderate-to-severe acute respiratory distress syndrome, consider using a higher positive end-expiratory pressure (PEEP) strategy (PEEP > 10 cm H2O) over a lower PEEP strategy.**
<u>Remark</u>	*This is a <u>low priority</u> recommendation and we do not expect to update it in the immediate future, however we continue to conduct daily searches for new evidence.*
**Prone positioning for adults**	
**Consensus recommendation**	**For mechanically ventilated adults with COVID-19 and hypoxaemia despite optimising ventilation, consider prone positioning for more than 12 hours a day.**
<u>Remark</u>	Current reports suggest prone ventilation is effective in improving hypoxia associated with COVID-19. This should be done in the context of a hospital guideline that includes suitable personal protective equipment (PPE) for staff and which minimises the risk of adverse events, e.g. accidental extubation.<br><br>Net clinical benefit for each patient should be considered on a case-by-case basis, as factors such as frailty, advanced illness or comorbidity may lessen the benefit and increase potential harms.

	Decisions around proceeding to more invasive forms of therapy should consider the preferences and values of the patient and whether they have an advanced care directive or plan, and should be discussed with the patient or their substitute / medical treatment decision-maker.<br><br>*This is a <u>low priority</u> recommendation and we do not expect to update it in the immediate future, however we continue to conduct daily searches for new evidence.*
**Conditional recommendation**<br><br><br><br><br><br>***<u>Remark</u>***	**For adults with COVID-19 and respiratory symptoms who are receiving any form of supplemental oxygen therapy and have not yet been intubated, consider prone positioning for at least 3 hours per day as tolerated. When positioning a patient in prone, ensure proning is used with caution and accompanied by close monitoring of the patient. Use of prone positioning should not delay endotracheal intubation and mechanical ventilation in patients with COVID-19 who are deteriorating despite optimised less invasive respiratory therapies.**<br><br>For adults with COVID-19 and respiratory symptoms who are receiving any form of supplemental oxygen therapy and have not yet been intubated, prone positioning for as long as tolerated may increase benefits.<br><br>Vulnerable people who are treated outside the ICU, for example people who are older and living with frailty, cognitive impairment or unable to communicate, may especially be at increased risk of harm from proning. Despite the potential risks of awake proning associated with frailty, there may be benefits for this group. The net clinical benefit for each individual patient should be considered on a case-by-case basis.<br><br>Currently, the evidence indicates that prone positioning probably decreases treatment failure and the need for intubation, with no increase in harms. Prone positioning should be done in the context of a hospital guideline that includes suitable personal protective equipment (PPE) for staff and which minimises the risk of adverse events.<br><br>*This is a <u>low priority</u> recommendation and we do not expect to update it in the immediate future, however we continue to conduct daily searches for new evidence.*
**Prone positioning and CPR**	
**Consensus recommendation**<br><br><br><br><br>***<u>Remark</u>***	**For patients with COVID-19 in prone position requiring cardiopulmonary resuscitation (CPR), where safe and feasible, return the patient to supine position and commence resuscitation.**<br>**If returning the patient to supine position is not safe and feasible, commence CPR in prone position. Once it is safe and feasible, return the patient to supine position and continue the resuscitation process.**<br><br>When caring for patients with COVID-19, consider the options available for providing cardiopulmonary resuscitation (CPR) when instituting prone positioning.

	It is reasonable to provide CPR in the prone position when supine CPR cannot be feasibly or safely implemented, and the airway is secured.<br><br>Returning the patient to supine position should only be performed when there are suitable resources to minimise risk of harm to staff and patients, e.g. accidental extubation, venous or arterial line dislodgement.<br><br>Provision of CPR for patients in prone position should be performed where there are hospital guidelines, and training in provision of prone CPR has been undertaken.<br><br>Decisions to commence CPR should consider the preferences and values of the patient and whether they have an advanced care directive or plan, and should be discussed with the patient or their substitute / medical treatment decision-maker in advance of need.<br><br>Consideration should also be given to whether prone positioning has contributed to the need for CPR, for example by including abdominal compression and obstruction to venous return.
**Recruitment manoeuvres**	
**Consensus recommendation**<br><br><br>***<u>Remark</u>***	**For mechanically ventilated adults with COVID-19 and hypoxaemia despite optimising ventilation, consider using recruitment manoeuvres.**<br>**If recruitment manoeuvres are used, do not use staircase or stepwise (incremental PEEP) recruitment manoeuvres.**<br><br>*This is a <u>low priority</u> recommendation and we do not expect to update it in the immediate future, however we continue to conduct daily searches for new evidence.*
**Extracorporeal membrane oxygenation (ECMO) for adults**	
**Conditional recommendation**<br><br><br>***<u>Remark</u>***	**Consider early referral to an ECMO centre for patients developing refractory respiratory failure in mechanically ventilated adults with COVID-19 (despite optimising ventilation, including proning and neuromuscular blockers).**<br><br>Due to the resource-intensive nature of ECMO and the need for experienced centres, healthcare workers and infrastructure, ECMO should only be considered in selected patients with COVID-19 and severe ARDS.<br><br>Net clinical benefit for each patient should be considered on a case-by-case basis, as factors such as frailty, advanced illness or comorbidity may lessen the benefit and increase potential harms.<br><br>Decisions around proceeding to more invasive forms of therapy should consider the preferences and values of the patient and whether they have an advanced care directive or plan, and should be discussed with the patient or their substitute / medical treatment decision-maker.<br><br>*This is a <u>low priority</u> recommendation and we do not expect to update it in the immediate future, however we continue to conduct daily searches for new evidence.*

ARDS – acute respiratory distress syndrome; FiO_2 – Fraction of inspired oxygen; ICU – intensive care unit

Table 6. Additional supportive recommendations for the treatment of individuals with COVID-19 (guideline sections 10.1, 11 and 13)

**Venous thromboembolism (VTE) prophylaxis for adults**	
**Conditional recommendation**	**Use prophylactic doses of anticoagulants, preferably low molecular weight heparin (LMWH) (e.g. enoxaparin 40 mg once daily or dalteparin 5000 IU once daily), in adults with moderate, severe or critical COVID-19 or other indications, unless there is a contraindication, such as risk for major bleeding. Where the estimated glomerular filtration rate (eGFR) (see below) is less than 30 mL/min/1.73m^2, unfractionated heparin or clearance-adjusted doses of LMWH may be used (e.g. enoxaparin 20 mg once daily).**
Remark	For body weights outside 50–90 kg or heights outside 150–180 cm, calculate the body surface area (BSA) and multiply the eGFR by BSA/1.73.<br><br>The Taskforce notes that in critical illness, creatinine-based estimation of kidney function can be unreliable.<br><br>*This is a moderate priority recommendation and will be updated when new evidence becomes available that is likely to impact the direction or strength of the recommendation.*
**Conditional recommendation against**	**Do not routinely offer therapeutic anticoagulant dosing in adults with moderate, severe or critical COVID-19. There is no additional indication for therapeutic dosing for anticoagulants in adults with severe or critical COVID-19 beyond current standard best practice.**
Remark	*This is a moderate priority recommendation and will be updated when new evidence becomes available that is likely to impact the direction or strength of the recommendation.*
**ACEIs/ARBs in patients with COVID-19**	
**Recommended**	**In patients with COVID-19 who are receiving ACEIs/ARBs, there is currently no evidence to deviate from usual care and these medications should be continued unless contraindicated.**
Remark	Stopping these medications abruptly can lead to acute heart failure or unstable blood pressure.<br><br>*This is a low priority recommendation and we do not expect to update it in the immediate future, however we continue to conduct daily searches for new evidence.*
**Steroids for people with asthma or COPD with COVID-19**	
**Consensus recommendation**	**Use inhaled or oral steroids for the management of people with co-existing asthma or chronic obstructive pulmonary disease (COPD) and COVID-19 as you would normally for viral exacerbation of asthma or COPD. Do not use a nebuliser.**
Remark	*This is a low priority recommendation and we do not expect to update it in the immediate future, however we continue to conduct daily searches for new evidence.*

Oestrogen-containing therapies in patients with COVID-19	
**Consensus recommendation**	**Consider stopping oral menopausal hormone therapy (MHT), also known as hormone replacement therapy (HRT), in women with mild or moderate COVID-19.**<br>**Before restarting oral MHT, review the indication for this. If MHT is continued, consider using a transdermal preparation.**
Remark	Decisions around stopping hormone therapy should be discussed with the patient or their substitute / medical treatment decision-maker. The goals of patient care need to balance the preferences and values of the patient, based on discussion and consideration of the patients individual circumstances.<br><br>*This is a moderate priority recommendation and will be updated when new evidence becomes available that is likely to impact the direction or strength of the recommendation.*
**Consensus recommendation**	**Stop oral menopausal hormone therapy (MHT) in women with severe or critical COVID-19.**<br>**Before restarting oral MHT, review the indication for this and consider transitioning to a transdermal preparation.**
Remark	Decisions around stopping hormone therapy should be discussed with the patient or their substitute / medical treatment decision-maker. The goals of patient care need to balance the preferences and values of the patient, based on discussion and consideration of the patients individual circumstances.<br><br>*This is a moderate priority recommendation and will be updated when new evidence becomes available that is likely to impact the direction or strength of the recommendation.*
**Consensus recommendation**	**In women who have COVID-19 and who are taking oestrogen-containing contraception, manage these medications as per usual care.**<br>**In women who stop or suspend contraception when they have COVID-19, restart contraception at the time of discharge or when acute symptoms have resolved.**
Remark	Decisions around stopping oestrogen-containing contraception should be discussed with the patient or their substitute / medical treatment decision-maker. The goals of patient care need to balance the preferences and values of the patient, based on discussion and consideration of the patients individual circumstances.<br><br>*This is a moderate priority recommendation and will be updated when new evidence becomes available that is likely to impact the direction or strength of the recommendation.*

Timing of surgery following COVID-19 infection	
**Conditional recommendation against**	**Do not routinely perform elective surgery within 7 weeks of recovery from acute illness, following a diagnosis of SARS-CoV-2 infection, unless outweighed by the risk of deferring surgery, such as disease progression or clinical priority.**
Remark	Informed consent and, where deemed necessary, shared decision-making with a valid substitute decision-maker, should include discussion about the potential increased risk of surgery following a diagnosis of COVID-19 and in the presence of post-acute COVID-19 symptoms.<br><br>*This is a moderate priority recommendation and will be updated when new evidence becomes available that is likely to impact the direction or strength of the recommendation.*
**Conditional recommendation**	**For people undergoing elective surgery following a diagnosis of SARS-CoV-2 infection, consider carrying out multisystem preoperative assessment in consultation with a unit familiar with the assessment of people recovering from COVID-19.**
Remark	*This is a moderate priority recommendation and will be updated when new evidence becomes available that is likely to impact the direction or strength of the recommendation.*

ACEIs/ARBs – angiotensin-converting enzyme (ACE) inhibitors / angiotensin II receptor blockers; SARS-CoV-2 – severe acute respiratory syndrome coronavirus 2

References

1. RECOVERY Collaborative Group. Casirivimab and imdevimab in patients admitted to hospital with COVID-19 (RECOVERY): a randomised, controlled, open-label, platform trial. Lancet 2022; 399: 665-676
2. National SARS-CoV-2 Serology Assay Evaluation Group. Performance characteristics of five immunoassays for SARS-CoV-2: a head-to-head benchmark comparison. Lancet Infect Dis 2020; 20: 1390-1400.
3. Somersan-Karakaya S, Mylonakis E, Menon V. Casirivimab and imdevimab for treatment of hospitalized patients with Covid-19. medRxiv 2021. https://doi.org/10.1101/2021.11.05.21265656
4. RECOVERY Collaborative Group. Baricitinib in patients admitted to hospital with COVID-19 (RECOVERY): a randomised, controlled, open-label, platform trial and updated meta-analysis. medRxiv 2022. https://doi.org/10.1101/2022.03.02.22271623
5. Kalil AC, Patterson TF, Mehta AK et al. Baricitinib plus remdesivir for hospitalized adults with Covid-19. N Eng J Med 2020; 384: 795-807
6. Marconi VC, Ramanan AV, de Bono S et al. Efficacy and safety of baricitinib for the treatment of hospitalised adults with COVID-19 (COV-BARRIER): a randomised, double-blind, parallel-group, placebo-controlled phase 3 trial. Lancet Resp Med 2021; 9: 1407-1418
7. Therapeutic Goods Administration. Shortage of tocilizumab (Actemra) medicines: Resolved. https://www.tga.gov.au/alert/shortage-tocilizumab-actemra-medicines-resolved (viewed Aug 2022)
8. RECOVERY Collaborative Group. Tocilizumab in patients admitted to hospital with COVID-19 (RECOVERY): a randomised, controlled, open-label, platform trial. Lancet 2021; 397: 1637-1645
9. REMAP-CAP Investigators, Gordon AC, Mouncey PR et al. Interleukin-6 receptor antagonists in critically ill patients with Covid-19. N Eng J Med 2021; 384: 1491-1502
10. Agarwal A, Rochwerg B, Lamontagne F. Update to living WHO guideline on drugs for covid-19. BMJ 2020; 371: m4475
11. Australian National COVID-19 Clinical Evidence Taskforce. Remdesivir methods brief. https://files.magicapp.org/guideline/256b4be2-a48d-4fc5-a86d-a92fe3232279/files/Remdesivir_Methods_Brief_r299401.pdf. (viewed Aug 2022)
12. Roche. Ronapreve does not retain neutralising activity against the Omicron variant. 16 December 2021. https://assets.cwp.roche.com/f/126832/x/db977d9333/2021216_roche-statement-on-ronapreve-omicron.pdf (viewed Aug 2022)
13. Norton T, Ali S, Sivapalasingam S, et al. REGEN-COV Antibody Combination in Outpatients With COVID-19 – Phase 1/2 Results. medRxiv 18 March 2022.
14. Weinreich DM, Sivapalasingam S, Norton T et al. REGEN-COV antibody combination and outcomes in outpatients with Covid-19. N Eng J Med 2021; 385: e81.
15. Yu L-M, Bafadhel M, Dorward J et al. Inhaled budesonide for COVID-19 in people at high risk of complications in the community in the UK (PRINCIPLE): a randomised, controlled, open-label, adaptive platform trial. Lancet 2021; 398: 843-855.
16. Jayk Bernal A, Gomes da Silva MM, Musungaie DB et al : Molnupiravir for oral treatment of Covid-19 in nonhospitalized patients. N Eng J Med 2022; 386: 509-520.
17. Therapeutic Goods Administration. Australian Product Information – Lagevrio® (molnupiravir) Capsules. https://www.tga.gov.au/sites/default/files/lagevrio-pi.pdf (viewed Aug 2022)
18. Tippabhotla SK, Lahiri S, Raju R. et al. Efficacy and safety of molnupiravir for the treatment of non-hospitalized adults with mild COVID-19: A randomized, open-label, parallel-group phase 3 trial. SSRN 2022, https://papers.ssrn.com/sol3/papers.cfm?abstract_id=4042673 (viewed Aug 2022)
19. Australian Government, Department of Health and Aged Care. The Pharmaceutical Benefits Scheme (Molnupiravir). https://www.pbs.gov.au/medicine/item/12910L (viewed Aug 2022)

20. National Centre for Immunisation Research and Surveillance (NCRIS): Zoster vaccines: Frequently asked questions. July 2021. URL https://www.ncirs.org.au/sites/default/files/2021-07/Zoster%20vaccines%20-%20Frequently%20asked%20questions_19%20July%202021_Final.pdf. (viewed Aug 2022)
21. Hammond J, Leister-Tebbe H, Gardner A et al. Oral nirmatrelvir for high-risk, nonhospitalized adults with Covid-19. N Eng J Med 2022 386: 1397-1408.
22. United States Food and Drug Administration. Fact Sheet for Healthcare Providers: Emergency Use Authorization for Paxlovid. https://www.fda.gov/media/155050/download (viewed Aug 2022)
23. University of Liverpool – COVID-19 Drug Interactions. https://www.covid19-druginteractions.org/checker (viewed Aug 2022)
24. Therapeutic Goods Administration. Australian Product Information – Paxlovid™ (nirmatrelvir/ritonavir tablets). https://www.tga.gov.au/sites/default/files/paxlovid-pi.pdf (viewed Aug 2022)
25. Australian Government, Department of Health and Aged Care. The Pharmaceutical Benefits Scheme (Nirmatrelvir (&) Ritonavir). https://www.pbs.gov.au/medicine/item/12996B (viewed Aug 2022)
26. Eom J, Ison M, Streinu-Cercel A. Efficacy and safety of CT-P59 plus standard of care: a phase 2/3 randomized, double-blind, placebo-controlled trial in outpatients with mild-to-moderate SARS-CoV-2 infection. Research Square 15 March 2021. https://doi.org/10.21203/rs.3.rs-296518/v1
27. Celltrion Inc, Incheon, Korea. Day 28 Clinical Study Report (Part 2), protocol number CT-P59 3.2 [unpublished].
28. Gottlieb RL, Vaca CE, Paredes R et al. Early remdesivir to prevent progression to severe Covid-19 in outpatients. N Eng J Med 2022; 386: 305-315.
29. Gupta A, Gonzalez-Rojas Y, Juarez E et al. Effect of sotrovimab on hospitalization or death among high-risk patients with mild to moderate COVID-19: a randomized clinical trial. JAMA 2022; 327: 1236-1246.
30. Montgomery H, Hobbs FDR, Padilla F et al. Efficacy and safety of intramuscular administration of tixagevimab-cilgavimab for early outpatient treatment of COVID-19 (TACKLE): a phase 3, randomised, double-blind, placebo-controlled trial. Lancet Resp Med 2022; S2213-2600: 00180-1
31. AstraZeneca Pharmaceuticals LP. Initial emergency use authorization (EUA) request for Evusheld (tixagevimab plus cilgavimab; AZD7442), for the prophylaxis of COVID-19. [unpublished]
32. Levin MJ, Ustianowski A, De Wit S et al. Intramuscular AZD7442 (tixagevimab-cilgavimab) for prevention of Covid-19. N Eng J Med 2022; 386: 2188-2200
33. O'Brien MP, Forleo-Neto E, Musser BJ et al : Subcutaneous REGEN-COV antibody combination to prevent Covid-19. N Eng J Med 2021; 385: 1184-1195

Acknowledgements

We would like to acknowledge all individual current and previous members of the COVID-19 Taskforce:

Steering Committee

Caroline Homer (Chair), Sharon McGowan (Past Chair), Nicola Ballenden, John Allan, Chris Cokis, Asha Bowen, Zoe Bradfield, Steven Faux, Terri-Lee Barrett, Vanessa Beavis, James Beckford Saunders, Tanya Buchanan, Marina Buchanan-Grey, Dawn Casey, Marita Cowie, Joseph Doyle, Mark Frydenberg, Danijela Gnjidic, Sally Green, Rohan Greenland, Ken Griffin, Stephan Groombridge, Anita Hobson-Powell, Louise Hardy, Alison Hodak, Ryan Lovett, Anthony Holley, Vase Jovanovska, Sabina Knight, Robert Lee, Kristin Michaels, Peter Morley, Andrew Miller, Julia Morphet, Sean Mutchmor, Suzi Nou, Linda Ng, Camilla Rowland, Phillip Russo, Megan Sarson, Hayley See, Alan Young, Veronica Le Nevez, Anoop Enjeti, Rod Mitchell, Gian Sberna, Nicola Lewis, Jason Soon, Louise Hardy, Fatima Mehmedbegovic.

Executive Team

Steve McGLoughlin, Tari Turner, Eloise Hudson, Sharon Gurry, Claire Beecher, Elaine Chong, Declan Primmer, Kayla Voss, Amy Moore, Julian Elliott, Rhiannon Tate, Britta Tendal, Sarah Norris, Bronwyn Morris-Donovan, Joshua Vogel, Shauna Hurley, Samantha Timms, Susan Whicker.

National Guidelines Leadership Group

Steve McGLoughlin (Co-Chair), Sutapa Mukherjee (Co-Chair), Ming Chen (Deputy Chair), Zoe Adey-Wakeling, Meera Agar, Jason Agostino, Bridget Barber, Karen Booth, Asha Bowen, Lucy Burr, Melissah Burnett, Katherine Isbister, Peter Cameron, Brendan Carrigan, Megan Cooper, Allen Cheng, Frances Dark, Rosalind Elliott, John Ferguson, Peter Fowler, Mark Frydenberg, Alan Glanville, Janeane Harlum, Sarah Hilmer, Carol Hodgson, Caroline Homer, Eleanor Horton, Brendan McMullan, Brett Mitchell, Mark Morgan, Christine Mott, Paul Myles, Vasi Naganathan, Priya Nair, Marty Nichols, Chris O'Donnell, Michael Parr, Pieter Peach, Vijay Roach, Wayne Varndell, Lyn Byers, Ewen McPhee, Jane Phillips, Julian Eliott (Past Co-Chair), Phillipa Hore, Ian Whyte, Leeroy William.

Consumer Panel

Rebecca Randall (Co-Chair), Eleanor Horton (Co-Chair), Chris Lassig, Joanne Muller, Sophie Noble, Nicholas Wood, Michelle King, Puspa Sherlock, Irene Mewburn, Kealey Griffiths, Richard Brightwell, Amrita Deshpande, Adam Ehm, Monica Ferrie, Satrio Nindyo Istiko, Lara Pullin, Elizabeth Robinson, Adele Witt, Lynda Condon, Elle Peters-Wisniak.

Disease-Modifying Treatment and Chemoprophylaxis Panel

Bridget Barber (Co-Chair), Josh Davis (Co-Chair), Megan Rees (Deputy Chair), Theresa Cole, Jane Davies, Dan Ewald, Amanda Gwee, Karin Leder, Gail Matthews, James McCarthy, James McMahon, Trisha Peel, Chris Raftery, Jason Roberts, Sarah Sasson, Ian Seppelt, Tom Snelling, Brad Wibrow, Katie Flanagan, Elena Shneider-Futschik, Michelle Giles.

Primary and Chronic Care Panel

Nicole Allard (Co-Chair), Mark Morgan (Co-Chair), Georgina Taylor (Deputy Chair), Kathryn Bailey, Penny Burns, Michelle Mason, Ben Chen, Steve Kisely, Sabina Knight, Sarah Larkins, Alistair Miller, Carmel Nelson, Bernard Shiu, Mieke van Driel, David Watters, Dina Watterson, Lyn Byers, Jason

Agostino, Paul Burgess, Kirsty Douglas, Ben Ewald, Dan Ewald, Dianna Fornasier, Lous Peachey, David Peiris, Rhonda Stuart, Lucie Walters, Ineke Weaver.

Acute and Critical Care Panel

Lucy Burr (Co-Chair), Priya Nair (Co-Chair), Simon Hendel (Deputy Co-Chair), Carol Hodgson (Deputy Co-Chair), Kiran Shekar (Deputy Co-Chair), Melissa Ankravs, Bronwyn Avard, Kelly Cairns, Craig French, Allan Glanville, Nicky Gilroy, Kim Hansen, Sue Huckson, Jon Iredell, Carrie Janerka, Rose Jaspers, Ed Litton, Stephen Macdonald, Steve McGloughlin, Paul Myles, Robert O'Sullivan, Sandra Peake, Owen Robinson, Chantel Sharland, Ian Seppelt, Peter Wark, Katherine Gibney, Phan Nguyen, Andrew Udy, Matt O'Sullivan, Huyen Tran, Sally McCarthy.

Pregnancy and Perinatal Care Panel

Caroline Homer (Co-Chair), Vijay Roach (Co-Chair), Michelle Giles (Deputy Co-Chair), Claire Whitehead (Deputy Co-Chair), Wendy Burton, Teena Downton, Glenda Gleeson, Adrienne Gordon, Karen Hose, Jenny Hunt, Nolan McDonnell, Philippa Middleton, Kellie Wilton, Antonia Shand, Jackie Kitschke, Jeremy Oats.

Paediatric and Adolescent Care Panel

Asha Bowen (Co-Chair), Brendan McMullan (Co-Chair), David Tingay (Deputy Co_Chair), Nan Vasilunas (Deputy Co-Chair), Lorraine Anderson, James Best, Penny Burns, Simon Craig, Dianne Crellin, Simon Erickson, Nick Fancourt, Zoy Goff, Vimbai Kapuya, Mark Moore, Danielle Wurzel, Luregn Schlapbach, Kathryn Roberts, Lorelle Maylon, Catherine Keyte.

Care of Older People and Palliative Care Panel

Meera Agar (Co-Chair), Richard Lindley (Co-Chair), Natasha Smallwood, Mandy Callary, Michael Chapman, Philip Good, Peter Jenkin, Deidre Morgan, Vasi Naganathan, Velandai Srikanth, Penny Tuffin, Elizabeth Whiting, Leeroy William, Patsy Yates, Rhonda Stuart. Expert Advisory Group: Ross Baker, Deborah Bateson, Christina Boros, David Burgner, Theresa Cole, Jennifer Curnow, Briony Cutts, Miles Davenport, Anoop Enjeti, Martha Hickey, Prahlad Ho, Adam Holyoak, Andrew Kelly, Helen Liley, James McFayden, Catriona Melville, Eileen Merriman, Helen Savoia, Magdalena Simonis, Gilda Tachedjian, Che Wee Tan, Huyen Tran, Chris Ward, Katrina Williams.

Pathways to Care Working Group

Penny Burns, Kim Hansen, Carrie Janerka, Brendan McMullan, Alistair Miller, Mark Morgan, Marty Nichols, Andrew Stewardson.

Cardiac Arrest Working Group

Jason Acworth, Neil Ballard, Samantha Bendall, Neel Bhanderi, Lyn Byers, Simon Craig, Dan Ellis, Dan Ewald, Craig Fairley, Brett Hoggard, Minh Le Cong, Peter Morley, Priya Nair, Andrew Pearce.

In Vitro Working Group

Miles Davenport, Jodie McVernon, Eva Stadler, Kanda Subbarao, Stuart Turville, David Khoury, Paul Young.

COVID-19 Care Working Group

Jo Muller, Michelle King, Sophie Noble, Irene Mewburn, Rebecca Randall, Ed Litton, Rose Jaspers, Carrie Janerka.

Evidence Team

Heath White, Tari Turner, Steve McDonald, Ming Chen, Nila Dharan, David Fraile Navarro, Saskia Cheyne, Annie Synott, Samantha Chakraborty, Leila Cusack, Alex Poole, Jessie Hewitt, Shruti Nair, Britta Tendal, Joshua Vogel, Henriette Callesen, Sue Campbell, Jenny Ring, Agnes Wilson, Tanya Millard, Melissa Murano, David Tunnicliffe.

Infection Prevention and Control Panel (Jan-June 2021)

John Ferguson (Co-Chair), Phillipa Hore (Co-Chair), Kathy Dempsey (Deputy Co-Chair), Deborah Friedman (Deputy Co-Chair), Brett Mitchell (Deputy Co-Chair), Penny Burns, Kate Cole, Melanie Dicks, Bridget Ferguson, Briony Hazelton, Nicole King, Sally McCarthy, Steve McGloughlin, Cherylynn McGurgan, Malcolm Sim, Andrew Stewardson, Barry Tam.

Observational Data Working Group (April 2020 – Dec 2021)

David Henry (Co-Chair), Sallie Pearson (Co-Chair), Douglas Boyle, Kendal Chidwick, Wendy Chapman, Craig French, Chris Pearce, Tom Snelling.

Technology Advisory Group (April 2020 – Dec 2021)

Hugh Williams (Chair), Dan Draper, Kelvin Hill, Sarah Norris.

Independent Conflicts of Interest Committee

Lisa Bero (Chair), Quinn Grundy, Joel Lexchin, Barbara Mintzes.
